# A soluble truncated tau species related to cognitive dysfunction is elevated in the brain of cognitively impaired human individuals

**DOI:** 10.1038/s41598-020-60777-x

**Published:** 2020-03-02

**Authors:** Peng Liu, Benjamin R. Smith, Michelle L. Montonye, Lisa J. Kemper, Kailee Leinonen-Wright, Kathryn M. Nelson, LeeAnn Higgins, Candace R. Guerrero, Todd W. Markowski, Xiaohui Zhao, Ashley J. Petersen, David S. Knopman, Ronald C. Petersen, Karen H. Ashe

**Affiliations:** 1 Department of Neurology, University of Minnesota, Minneapolis, MN 55455 USA; 2 Department of Neuroscience, University of Minnesota, Minneapolis, MN 55455 USA; 3 N. Bud Grossman Center for Memory Research and Care, University of Minnesota, Minneapolis, MN 55455 USA; 4 Institute for Therapeutics Discovery and Development, University of Minnesota, Minneapolis, MN 55455 USA; 5 Department of Biochemistry, Molecular Biology, and Biophysics, University of Minnesota, St. Paul, MN 55108 USA; 6 Division of Biostatistics, University of Minnesota, Minneapolis, MN 55455 USA; 7 Department of Neurology, Mayo Clinic, Rochester, MN 55905 USA; 8 Geriatric Research, Education, and Clinical Centers, Veterans Affairs Medical Center, Minneapolis, MN 55417 USA; 9 Present Address: AstraZeneca, Gaithersburg, MD 20878 USA

**Keywords:** Biochemistry, Neuroscience

## Abstract

Neurofibrillary tangles are a pathological hallmark of Alzheimer’s disease, and their levels correlate with the severity of cognitive dysfunction in humans. However, experimental evidence suggests that soluble tau species cause cognitive deficits and memory impairment. Our recent study suggests that caspase-2 (Casp2)-catalyzed tau cleavage at aspartate 314 mediates synaptic dysfunction and memory impairment in mouse and cellular models of neurodegenerative disorders. Δtau314, the C-terminally-truncated cleavage products, are soluble and present in human brain. In addition, levels of Δtau314 proteins are elevated in the brain of the cognitively impaired individuals compared to the cognitively normal individuals, indicating a possible role for Δtau314 proteins in cognitive deterioration. Here we show that (1) Δtau314 proteins are present in the inferior temporal gyrus of human brains; (2) Δtau314 proteins are generated from all six tau splicing isoforms, (3) levels of both Casp2 and Δtau314 proteins are elevated in cognitively impaired individuals compared to cognitively normal individuals, and (4) levels of Δtau314 proteins show a modest predictive value for dementia. These findings advance our understanding of the characteristics of Δtau314 proteins and their relevance to cognitive dysfunction and shed light on the contribution of Casp2-mediated Δtau314 production to cognitive deterioration.

## Introduction

Tau is a soluble and unstructured protein, is enriched in neurons of the central nervous system (CNS), and plays an essential role in the formation and stabilization of microtubules to maintain normal neuronal structure and function^1,2^. In the human CNS, six isoforms of tau are generated by alternative splicing of exons 2, 3, or 10 of mRNA^3,4^. Tau is subjected to more than ten types of post-translational modifications (PTMs); under pathological conditions, the coordinated actions of multiple tau PTMs result in its dissociation from microtubules and intra-cellular aggregation to form neurofibrillary tangles (NFTs), a pathological hallmark of Alzheimer’s disease (AD) (for review, see^5^). As the burden of NFTs in a variety of brain regions correlate well with the severity of cognitive deficits of AD patients (for example, see^6–10^), NFTs were assumed to be one of the major drivers of cognitive impairment. However, Gomez-Isla and colleagues observed that in the brain of individuals with AD, both the amount of neuron loss and the amount of NFTs in the disease-affected superior temporal sulcus correlate with the duration and severity of cognitive dysfunction, but the former exceeds the latter more than 7-fold. This suggests that neuronal loss, rather than NFTs, contributes directly to cognitive dysfunction in AD^11^. Further, several studies using tau transgenic mouse models have shown that synaptic function impairment and cognitive deficits occur before or without the formation of NFTs, and that switching off tau expression ameliorates memory impairment even though NFTs remain^12–14^. In addition, NFT-bearing neurons in the visual cortex function normally in electrophysiological studies^15^. Taken together, these findings suggest that soluble tau species, but not NFTs, could be responsible for cognitive dysfunction.

A recent study identified Δtau314^16^ in the rTg4510 mouse line^12,17^ that overexpresses transgenic human tau 0N4R isoform with the proline-to-leucine mutation at amino acid residue 301 (P301L) associated with frontotemporal dementia and parkinsonism linked to chromosome 17^18,19^. Δtau314 are C-terminally-truncated, soluble tau species that are produced from caspase-2 (Casp2)-catalyzed cleavage at aspartate 314 (D314, tau 2N4R isoform numbering system hereafter unless specified). Lowering endogenous levels of Casp2 reduces Δtau314 production and ameliorates memory deficits in rTg4510 mice; whereas rendering tau non-cleavable by an aspartate-to-glutamate mutation at D314 (D314E) blocks tau mislocalization to dendritic spines and attenuates synaptic function impairment in cultured primary neurons expressing the tau P301L mutant^16^. Further, transduction of mice with adeno-associated virus (AAV) carrying this additional tau D314E mutation prevents cognitive deficits caused by tau P301L-carrying AAV-mediated transduction^16^. Together, these findings support Casp2-mediated tau cleavage at D314 as a pathogenic event that leads to cognitive dysfunction. In addition, the relevance of Δtau314 to AD was established in a cohort from the Memory and Aging Project at the Rush University, Chicago, IL^20^, in that levels of Δtau314 proteins are elevated in the disease-vulnerable inferior temporal gyrus (ITG) of the cognitively impaired (CI) individuals compared to the cognitively normal (CN) individuals^16^.

To confirm the existence of Δtau314 proteins in human brains and their relevance to cognitive dysfunction, we analyzed post-mortem ITG specimens in a cohort from the Mayo Clinic Study of Aging at the Mayo Clinic, Rochester, MN. This cohort consisted of ninety persons (twenty-four with amnestic dementia who had relatively pure AD pathology at post-mortem examination (AD dementia), thirty-three individuals who died while still being diagnosed with mild cognitive impairment (MCI) and who had at autopsy primarily AD pathology, and thirty-three individuals who were CN within 24 months before death (twenty-eight of them remaining CN within 18 months before death)). This cohort was analyzed using conventional immunoprecipitation (IP)/Western blotting (WB), combined with IP/mass spectrometry (MS) and an ultra-sensitive, enzyme-linked immunosorbent assay (ELISA). We found Δtau314 proteins derived from all six tau splicing isoforms in ITG and elevated levels of both Casp2 and Δtau314 proteins in the CI individuals (AD dementia and MCI) compared to the CN individuals. This study further characterizes Δtau314 proteins and their relationship to AD and cognition, and it confirms the finding that levels of Δtau314 proteins are elevated in CI individuals in a second cohort. These results contribute to our understanding of AD pathogenesis and development of biomarkers for synaptic deficits.

## Results

### Antibodies recognize Δtau314 with specificity

**Figure 1:**
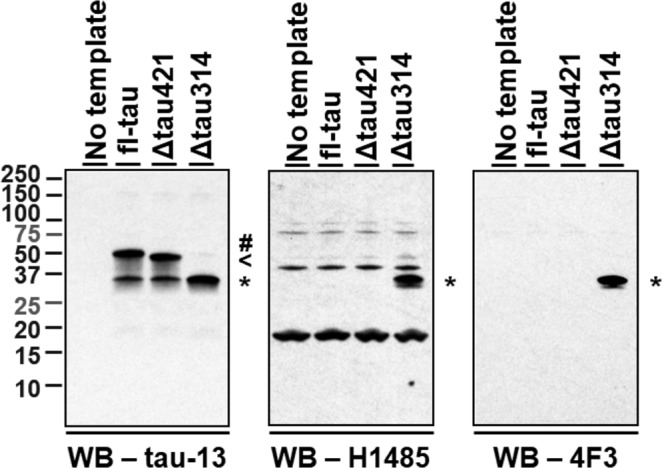
The antibodies H1485 and 4F3 recognize Δtau314 proteins with specificity. Three µL of the rabbit reticulocyte lysates containing synthetic full-length 0N4R tau (fl-tau), Δtau421, or Δtau314 proteins were subjected to Western blotting. The blots were probed by the monoclonal antibody tau-13 directed against an N-terminal epitope (a.a. 20–35) of human tau (left panel) and the polyclonal antibody H1485 (middle panel) and the monoclonal antibody 4F3 (right panel), both of which targets tau epitopes ending C-terminally at D314. While tau-13 detects fl-tau (hash mark), Δtau421 (arrowhead), and Δtau314 (asterisk), both H1485 and 4F3 recognize only Δtau314 (asterisks). Notably, proteins migrating similarly as Δtau314 in samples containing Δtau421 and fl-tau in the tau-13-probed blot may represent the tau proteins of the similar size to, but different from, Δtau314. Bands other than the ~35-kDa are likely non-specific, as all these bands appear in a sample in which no tau synthesis DNA template was included. The three full-length blots were prepared from three different 10–20% Tris-Tricine precast gels, and the same exposure time was applied to the development of the three blots. There was no grouping within each blot.

We first assessed the binding specificity of the H1485 and 4F3 antibodies developed to target the truncated tau proteins ending C-terminally at D314 (Table 1). For this, we synthesized the full-length human tau 0N4R splicing isoform (fl-tau) and Δtau421 and Δtau314, two truncated tau proteins ending C-terminally at aspartate 421 and D314, respectively. Both antibodies detected Δtau314, but not fl-tau or Δtau421, though the polyclonal H1485 also recognized several endogenous proteins present in the synthetic system (Fig. 1). The results thus indicate that both H1485 and 4F3 recognize Δtau314 with specificity.

**Table 1:** Antibodies used in this study.

Antibody	Host/Isotype	Epitope^a^	Source	Usage
Tau-13	Ms, IgG_1, κ_	Tau_15–25_	BioLegend Cat# 835201, RRID:AB_2565341	IP (10 µg); WB (1:30,000, final conc. n.d.)
Tau-5	Ms, IgG_1_	Tau_210–241_	Thermo Fisher Scientific Cat# AHB0042, RRID:AB_2536235	WB (1:30,000, 17 ng/mL)
Anti-3R	Ms, IgG	Tau_267–277&309–313_	MilliporeSigma Cat# 05-803, RRID:AB_11212693	WB (1:5,000, final conc. n.d.)
Anti-4R	Ms, IgG	Tau_275–291_	MilliporeSigma Cat# 05-804, RRID:AB_310014	WB (1:5,000, final conc. n.d.)
(biotin-conjugated) 4F3	Ms, IgG_2b, κ_	Tau_x−314_	Ashe laboratory	WB (1:45,000, 36 ng/mL); Capture reagent (ELISA)
H1485	Rb, IgG	Tau_x−314_	Ashe laboratory	IP (4 µg); WB (1:2,500, 280 ng/mL)
Biotin-conjugated HT7	Ms, IgG_1, κ_	Tau_159–163_	Thermo Fisher Scientific Cat# MN1000B, RRID:AB_223453	Detection reagent (ELISA)
Biotin-conjugated BT2	Ms, IgG_1, κ_	Tau_194–198_	Thermo Fisher Scientific Cat# MN1010B, RRID:AB_10974155	Detection reagent (ELISA)
Q_C_	proprietary	Tau_218–222_	Quanterix, Cat. #: unlisted	Capture reagent (ELISA)
Q_D_	proprietary	Tau_16–24_	Quanterix, Cat. #: unlisted	Detection reagent (ELISA)
Anti-βIII-tubulin	Ms, IgG_2b_	C-terminus	MilliporeSigma Cat# T8578, RRID:AB_1841228	WB (1:200,000, 5 ng/mL)
Anti-Casp2	Rb, IgG	C-terminus	Abcam, Cat. #: 179519	WB (1:5,000, 496 ng/mL)

^a^Amino acid residues of tau protein are counted using the 2N4R splicing isoform numbering system.

Ms = mouse, Rb = rabbit, conc. = concentration, n.d. = not determined.

### Δtau314 proteins are present in post-mortem human brains

**Figure 2:**
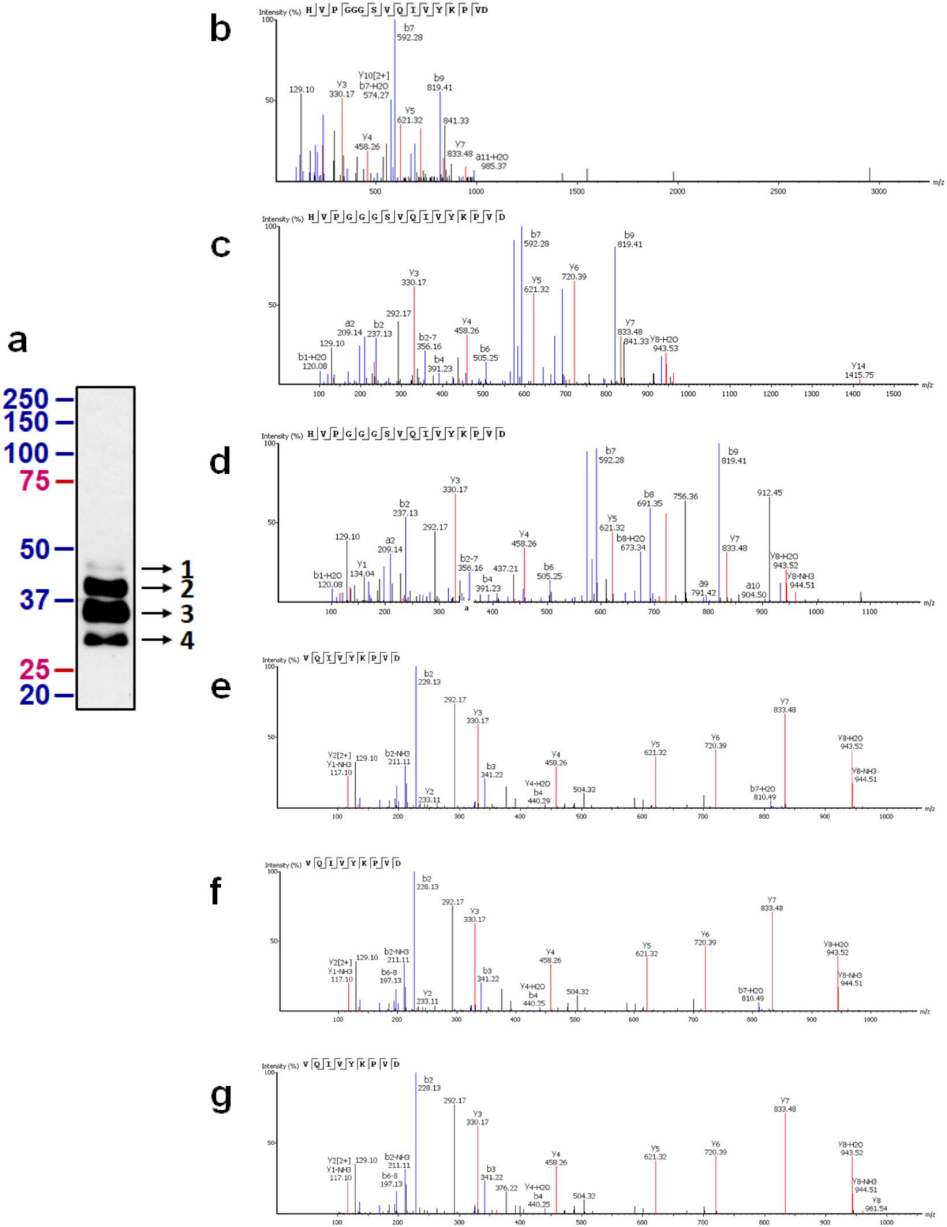
Δtau314 proteins in the inferior temporal gyrus (ITG) are identified using immunoprecipitation coupled to mass spectrometry. (**a**) An H1485-probed Western blot of eluates of the tau-13-immunoprecipitated brain proteins reveals the protein bands (arrows with the numbers 1–4) that were subjected to mass spectrometry (MS) analysis. (**b**–**d**) MS/MS spectra showing in bands 1 (**b**), 2 (**c**), and 3 (**d**) the identification of the peptide HVPGGGSVQIVYKPVD that resides only in the 4R-tau isoforms. (**e**–**g**) MS/MS spectra showing in bands 2 (**e**), 3 (**f**), and 4 (**g**) the identification of the peptide VQIVYKPVD that resides only in the 3R-tau isoforms.

We asked whether Δtau314 proteins are present in the ITG of human brains. We performed IP/WB analysis of post-mortem specimens to detect the tau-13-immunoprecipitated proteins that are reactive to H1485 or the biotin-conjugated 4F3 antibody (Table 1). We identified protein bands that electrophoretically migrated at ~45-, ~40-, ~35-, and ~30- kilo-Dalton (kDa) (Fig. 2a), potentially representing Δtau314 proteins that are produced from six tau splicing isoforms. To confirm this, we performed liquid chromatography (LC)-tandem MS (MS/MS) analysis of the trypsin-digested peptides extracted from gel pieces containing the H1485-reactive proteins. We identified the peptide HVPGGGSVQIVYKPVD, the C-terminal fragment of Δtau314 proteins specifically produced from the tau isoforms harboring four microtubule-binding motifs (4R-tau), from the gel pieces containing the ~45-, ~40-, and ~35- kDa proteins (Fig. 2b–d, Supplementary Figs. S1 and S2); in parallel, we identified the peptide VQIVYKPVD, the C-terminal fragment of Δtau314 proteins produced from the tau isoforms harboring three microtubule-binding motifs (3R-tau), from the gel pieces containing the ~40-, ~35-, and ~30- kDa proteins (Fig. 2e–g, Supplementary Figs. S1 and S2**)**.

**Figure 3:**
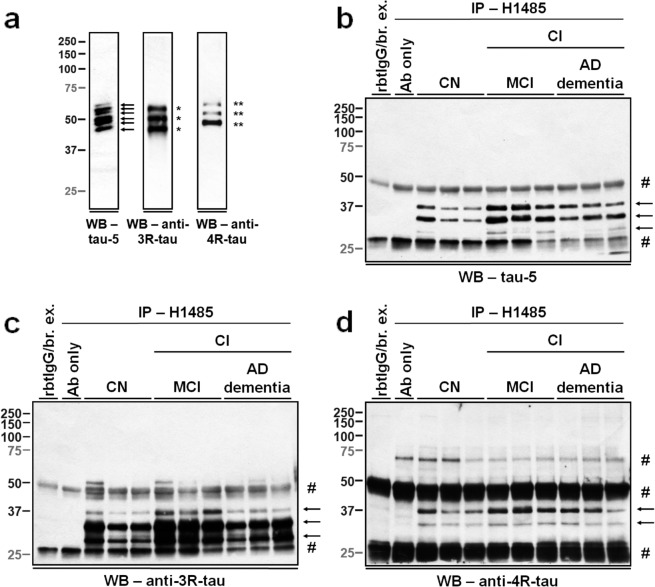
Δtau314 proteins are produced from both the 4R- and the 3R- tau isoforms. (**a**) A tau-13-probed Western blot (left panel) of the six purified recombinant human tau proteins shows all the six isoforms (arrows). Using an anti-3R tau antibody (middle panel), three of the six tau-13-reactive bands (single asterisks) were detected, representing the three 3R-tau isoforms. In parallel, an anti-4R tau antibody (right panel) detected the other three tau-13-reactive bands (double asterisks), representing the three 4R-tau isoforms. The three full-length blots were prepared from three different 10% Tris-HCl precast gels, and the same exposure time was applied to the development of the three blots. (**b**–**d**) Immunoprecipitation/Western blotting (IP/WB) analyses show that the H1485-immunoprecipitated Δtau314 proteins from representative samples (three cognitively normal (CN) individuals and six cognitively impaired (CI) individuals including three individuals with mild cognitive impairment (MCI) and three Alzheimer’s disease dementia patients (AD dementia), randomly selected within each group, and 300 µg of proteins per sample) were detected by the biotin-conjugated tau-5 antibody that recognizes all six human tau isoforms (**b**), the anti-4R antibody (**c**), and the anti-3R tau antibody (**d**) (arrows), respectively. Hash marks, non-specific bands. rbtIgG/br. ex., rabbit IgG-immunoprecipitated brain extracts. Ab only, H1485 antibody IP without brain extracts. These full-length blots were prepared from different 10% Tris-HCl precast gels, and different exposure times were applied to the development of the three blots to achieve the best signal-to-noise ratio for each blot. There was no grouping within each blot.

To further verify that Δtau314 proteins are products of all six tau isoforms, we immunoprecipitated the intracellularly-enriched (IC) fraction of ITG homogenates using H1485 and probed blots using monoclonal antibodies specifically targeting the 4R- and 3R- tau, respectively (Table 1). The binding specificities of antibodies to the 4R- and 3R- tau isoforms were verified (Fig. 3a). In randomly selected samples, we showed that the ~40-, ~35-, and ~30- kDa proteins were detected by the monoclonal tau-5 antibody directed against an epitope present in all six tau isoforms (Fig. 3b). The ~40-kDa proteins are likely comprised of the Δtau314 proteins that are produced from the 1N4R and 2N3R tau isoforms; the ~35-kDa, the 0N4R and 1N3R tau isoforms; and the ~30-kDa, the 0N3R tau isoform. The anti-3R-tau antibody detected all the three proteins (Fig. 3c), and the anti-4R tau antibody detected the ~40- and the ~35- kDa (Fig. 3d). We did not detect the ~45-kDa protein that presumably matches the Δtau314 protein produced from the 2N4R tau isoform using either tau-5 or anti-4R tau as the detection antibody. This failure is likely due to 1) the scarce level of this ~45-kDa Δtau314 protein present in the studied brain tissue and 2) detection interference from non-specific protein bands. Taken together, results of IP/WB substantiate the finding that Δtau314 proteins in human ITG are produced from both the 4R- and the 3R- tau isoforms.

### Levels of Δtau314 proteins are elevated in the brain of cognitively impaired individuals

We asked how levels of Δtau314 proteins differ in the AD-affected ITG of a cohort containing CI (including individuals with MCI and AD dementia patients who had no other pathologies other than plaques and tangles) and CN individuals (Supplementary Table S1). First, we compared the demographic and neuropathological features of the AD dementia, MCI, and CN individuals. We found that neither the sex distributions nor the post-mortem intervals (PMIs) differed among the three groups; however, the ages at death of the AD dementia patients were higher than the CN individuals by 6% (Table 2, Supplementary Fig. S3). As expected, these three different diagnostic groups are distinct in Braak stage neuropathology^21,22^ (Table 2, Supplementary Fig. S3) and neuritic plaque density^23^ (Table 2, Supplementary Fig. S3).

**Table 2:** Demographic and neuropathological characteristics of participants.

	CN^a^	MCI	AD dementia	*P* value^b^
Sample size, *N*	33	33	24	
Age at death [yr]:				
median (1^st^ quartile; 3^rd^ quartile)	87 (81,91)	89 (86.5, 92.5)	90.5 (87.5, 93)	0.022
range	61–94	75–103	82–99	
Sex: female/male, No. (% female)	19/14 (58)	15/18 (45)	17/7 (71)	0.160
Post-mortem interval [hr]:				
median (1^st^ quartile; 3^rd^ quartile)	12.8 (4.1, 18.4)	12 (7.75, 18.8)	12.25 (7, 20.88)	0.793
range	2–26	2–35	2–44	
Braak stage^c^:				<0.0001
Sample size, N (% within group)				
0	1 (3)	0 (0)	0 (0)	
1	4 (12)	1 (3)	0 (0)	
2	17 (52)	11 (33)	0 (0)	
3	8 (24)	5 (15)	2 (8)	
4	2 (6)	12 (36)	10 (42)	
5	0 (0)	4 (12)	9 (38)	
6	0 (0)	0 (0)	3 (13)	
CERAD-neuritic plaque density^d^:				<0.0001
Sample size, N (% within group)				
0	15 (45)	10 (30)	2 (8)	
1	11 (33)	4 (12)	4 (17)	
2	6 (18)	16 (48)	9 (38)	
3	0 (0)	3 (9)	9 (38)	

^a^One CN participant was missing values for Braak stage and CERAD plaque density.

^b^Comparisons were performed among cognitively normal (CN) individuals, individuals with mild cognitive impairment (MCI), and Alzheimer disease (AD) dementia patients using Kruskal-Wallis tests, except that for comparison of female proportions, a chi-square test was used. For *post hoc* comparison between pairs of groups, see Supplementary Fig. S3.

^c^Braak stage^21^ was assigned based on a procedure previously described^22^. Specifically, 0 = AD-type neurofibrillary degeneration not present, 1 = Braak stage I, 2 = Braak stage II, 3 = Braak stage III, 4 = Braak stage IV, 5 = Braak stage V, 6 = Braak stage VI.

^d^The consortium to establish a registry for Alzheimer’s disease (CERAD) score for density of neocortical neuritic plaque^23^ was assigned based on a procedure previously described^22^. Specifically, 0 = no neuritic plaques, 1 = sparse neuritic plaques, 2 = moderate neuritic plaques, 3 = frequent neuritic plaques.

**Figure 4:**
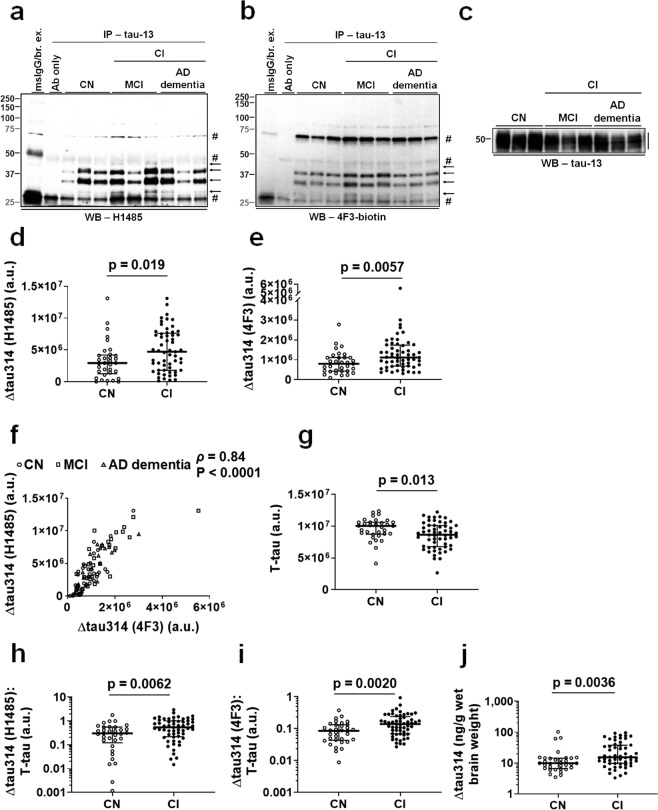
Levels of Δtau314 proteins are elevated in the cognitively impaired individuals. (**a**,**b**) Representative IP/WB showing that the tau-13-immunoprecipitated Δtau314 proteins (arrows) are detected by H1485 (**a**) and the biotin-conjugated 4F3 antibody (**b**). These two full-length blots were prepared from different 10% Tris-HCl precast gels, and different exposure times were applied to the development of the blots to achieve the best signal-to-noise ratio for each blot. There was no grouping within each blot. (**c**) Representative WB showing that the tau-13-immunoreactive proteins (the full-length blot is presented in Supplementary Figure S5. There was no grouping within this blot). (**d**,**e**) Comparison of levels of the H1485- (**d**) and the 4F3- reactive (**e**) proteins between the cognitively impaired (CI, *n* = 57) and the cognitively normal (CN, *n* = 33) individuals. (**f**) Correlation of levels of the H1485- versus the 4F3- reactive, tau-13-immunoprecipitated proteins. (**g**) Comparison of levels of the tau-13-reactive proteins between the CI and the CN individuals. (**h**,**i**) Comparison of levels of the H1485- (**h**) and the 4F3- reactive (**i**) proteins between the CI and the CN individuals following normalization to levels of the tau-13-reactive proteins. (**j**) Comparison of levels of the Δtau314 proteins determined using an ultra-sensitive ELISA between the CI (*n* = 56, one was missing due to shortage of sample supply) and the CN individuals (*n* = 33). Notably, the y-axes in figures (**h**–**j**) are in the log scale. For figures (**d**,**e**,**g**–**j**, Mann-Whitney tests were used; medians (middle long bars) and 1^st^ (lower short bars) and 3^rd^ (upper short bars) quartiles are shown. For figure (**f**), Spearman’s rank-order correlation was used. MCI, individuals with mild cognitive impairment; AD dementia, patients with Alzheimer’s disease dementia.

We then performed IP/WB to quantitatively analyze levels of Δtau314 proteins in the CI and the CN individuals. We showed that levels of Δtau314 proteins, detected by H1485 (Fig. 4a,d, Table 3) and the biotin-conjugated 4F3 (Fig. 4b,e, Table 3), in the CI individuals were 1.6- and 1.4- fold higher than in the CN individuals, respectively. Levels of Δtau314 proteins in the AD dementia patients and the individuals with MCI did not differ (Supplementary Fig. S4, Supplementary Table S2). Not surprisingly, levels of the H1485-detected Δtau314 proteins were highly correlated with levels of the 4F3-detected (Fig. 4f), indicating that these two antibodies share antigen-recognizing specificity.

**Table 3:** A statistical comparison of protein levels of cognitively impaired and cognitively normal individuals.

	Mann-Whitney	Two-tailed, unpaired *t*-test^a^	Multiple linear regression^a,b^
Δtau314 (4F3)	*p* =0.0057	*p* = 0.0051	*p* = 0.012
Δtau314 (H1485)	*p* =0.019	*p* = 0.026	*p* = 0.045
T-tau	*p* =0.013	*p* = 0.012	*p* = 0.053
Δtau314 (4F3):T-tau	*p =*0.0020	*p* = 0.0016	*p* = 0.0059
Δtau314 (H1485):T-tau	*p* =0.0062	*p* = 0.014	*p* = 0.027
βIII-tubulin	*p* = 0.076	*p* = 0.067	*p* = 0.13
Δtau314 (ELISA)	*p* =0.0036	*p* = 0.0039	*p* = 0.0094
T-tau (ELISA)	*p* = 0.80	*p* = 0.93	*p* = 0.82
Δtau314 (ELISA):T-tau (ELISA)	*p* = 0.16	*p* = 0.11	*p* = 0.17
Casp2:total proteins	*p* = 0.020	*p* = 0.040	*p* = 0.089

^a^The two-tailed, unpaired *t*-tests and multiple linear regressions were performed on the log-transformed outcomes.

^b^Multiple linear regression was used to analyze protein levels that were adjusted for age at death, sex, and PMI of brain tissue harvest of individuals.

Next, to compare the proportion of soluble total tau (T-tau) comprised of Δtau314 proteins between groups, we normalized levels of Δtau314 proteins to levels of T-tau proteins that are immunoreactive to tau-13. Interestingly, levels of T-tau proteins were 1.2-fold higher in the CN individuals than in the CI individuals (Fig. 4c,g, Supplementary Fig. S5, Table 3), and levels of T-tau proteins in the AD dementia patients and the individuals with MCI did not differ (Supplementary Fig. S4, Supplementary Table S2). The lower levels of T-tau proteins in the AD dementia patients and the individuals with MCI are unrelated to neuronal loss since levels of βIII-tubulin, a molecular proxy for neuron number, did not differ between the CN and the CI individuals (Table 3).

When normalized to levels of T-tau proteins, levels of H1485- and 4F3- reactive Δtau314 proteins in the CI individuals were 1.8- and 1.6- fold higher than in the CN individuals, respectively (Fig. 4h,i, Table 3). Levels of Δtau314 proteins in the AD dementia patients and the individuals with MCI were comparable (Supplementary Fig. S4, Supplementary Table S2).

Next, we performed protein quantification using Quanterix single molecule array (simoa), an ultra-sensitive ELISA. We showed that levels of the 4F3-captured, BT2/HT7-detected Δtau314 proteins (Table 1) were 1.5-fold higher in the CI individuals than in the CN individuals (Fig. 4j, Table 3), and levels of these Δtau314 proteins were comparable between the AD dementia patients and the individuals with MCI (Supplementary Fig. S4, Supplementary Table S2), which are consistent with the IP/WB findings. Notably, the Δtau314 protein species detected by the two methods may not be fully identical as N-terminally truncated Δtau314 proteins, if present, can also be included in the quantification using ELISA. We also quantified T-tau proteins using an antibody (Q_C_) targeting tau mid-region as the capture reagent and an antibody (Q_D_) targeting N-terminal tau as the detection reagent (Table 1). We showed that levels of T-tau proteins did not differ between the CN and the CI individuals (Table 3) or between the AD dementia patients and the individuals with MCI (Supplementary Table S2), which is discrepant to the results of IP/WB. This discrepancy may arise from differences in the capture and the detection antibody combination between IP/WB and ELISA (Table 1), which could result in measuring two different sets of tau proteins by the two methods. Not unexpectedly, levels of Δtau314 proteins, when normalized to levels of T-tau proteins, did not differ between the CN and the CI individuals (Table 3) or between the AD dementia patients and the individuals with MCI (Supplementary Table S2).

**Figure 5:**
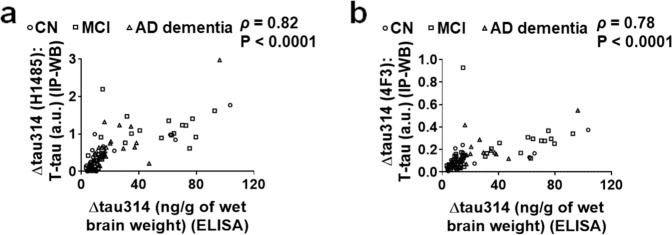
The correlation of levels of Δtau314 proteins determined by IP/WB versus ELISA. (**a**,**b**) Levels of the H1485- (**a**) and the 4F3- reactive (**b**) Δtau314 proteins (normalized to levels of the total tau (T-tau) proteins that are immunoreactive to tau-13) determined by IP/WB correlate to level of the Δtau314 proteins determined by ELISA. Spearman’s rank-order correlation analyses were used. CN, cognitively normal individuals; MCI, individuals with mild cognitive impairment; AD dementia, patients with Alzheimer’s disease dementia.

Further, we asked how levels of Δtau314 proteins measured by IP/WB (normalized to levels of T-tau proteins) correlate with levels of Δtau314 proteins measured by ELISA. We found that levels of Δtau314 proteins measured by the two methods correlated well (Fig. 5), despite the possibility that different Δtau314 proteins may be measured by the two experimental approaches.

We then asked whether the demographic and neuropathological features of our participants were associated with levels of Δtau314 proteins (normalized to levels of T-tau proteins). Interestingly, we identified that the normalized levels of Δtau314 proteins correlated with not only amyloid pathology densities (Supplementary Fig. S6), but also, to a lesser degree, Braak stages (Supplementary Fig. S7). This suggests a potential link between Δtau314 proteins and AD pathology. In addition, the ages at death of participants did not correlate with the normalized Δtau314 protein levels (Supplementary Fig. S8). The normalized levels of Δtau314 proteins were also comparable between the female and the male individuals (Supplementary Fig. S8). PMIs correlated notably with the normalized Δtau314 protein levels (Supplementary Fig. S8), indicating that PMI is likely a confounder that may affect levels of Δtau314 proteins in the studied participants.

We then reassessed levels of tau protein species following adjustment for age at death, sex, and PMI. When adjusted for the three demographic factors, levels of Δtau314 proteins measured by IP/WB, whether normalized to T-tau levels or not, remained higher in the CI individuals than in the CN individuals (Table 3). Thus, these data confirm that levels of Δtau314 proteins are elevated in the ITG of the CI individuals. Notably, the reassessed levels of Δtau314 proteins measured by ELISA, when normalized to T-tau levels, were no longer significantly different between the CI individuals and the CN individuals (Table 3). This is likely due to differences in T-tau species measured by ELISA versus IP/WB as discussed above.

### Levels of Δtau314 predicts cognitive impairment of individuals

**Figure 6:**
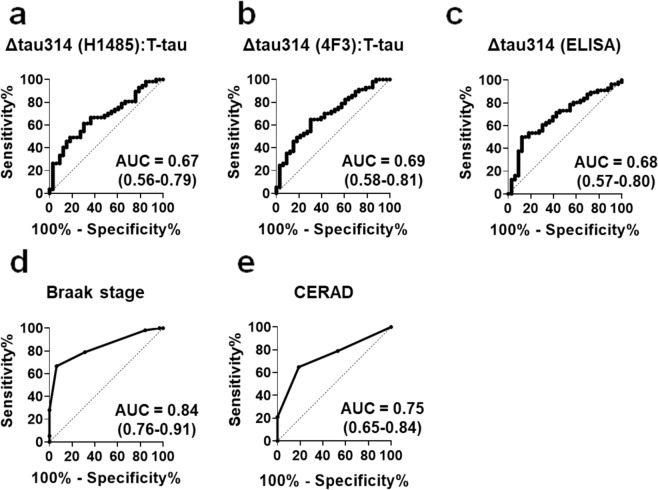
The receiver operating characteristic analyses of Δtau314, neurofibrillary tangle staging and neuritic plaque density in the cognitively impaired versus the cognitively normal cases. The receiver operating characteristic (ROC) curves of levels of the H1485- (**a**) and the 4F3- reactive (**b**) Δtau314 (normalized to levels of the soluble total tau (T-tau) proteins that are immunoreactive to tau-13), levels of the Δtau314 proteins determined by ELISA (**c**), neurofibrillary tangle staging (Braak stage, **d**), and neuritic plaque density (CERAD scores, **e**) are shown. The predictive values (*i.e*., area under a curve (AUC)) and 95% confidence intervals (in parentheses) of these parameters for cognitive impairment are shown. Dotted line is the line of identity (AUC = 0.5).

To assess the strength of the associations between cognitive impairment and Δtau314 proteins, we generated receiver operating characteristic (ROC) curves in which participants were categorized as CN or CI. The area under a curve (AUC) values were 0.67 and 0.69 for levels of H1485- (Fig. 6a) and 4F3- (Fig. 6b) immunoreactive Δtau314 (normalized to levels of T-tau), respectively, and the AUC value was 0.68 for levels of Δtau314 determined by ELISA (Fig. 6c). The AUC values for Braak staging (Fig. 6d) and neuritic plaque density (Fig. 6e) were 0.84 and 0.75, respectively. Statistical analyses of ROC curves showed that the AUC values of levels of Δtau314 proteins were lower than that of Braak staging but comparable to that of neuritic plaque density (Table 4).

**Table 4:** A statistical comparison of AUCs from ROC curves comparing cognitively impaired and cognitively normal individuals.

	Δtau314 (4F3):T-tau	Δtau314 (ELISA)	Braak Stage	CERAD
Δtau314 (H1485):T-tau	*p* = 0.48	*p* = 0.66	*p* = 0.023	*p* = 0.35
Δtau314 (4F3):T-tau		*p* = 0.90	*p* = 0.046	*p* = 0.51
Δtau314 (ELISA)			*p* = 0.043	*p* = 0.48
Braak Stage				*p* = 0.083

Area under a curve (AUC) values were compared using DeLong’s tests for correlated ROC curves.

### Levels of Casp2 are higher in the brain of cognitively impaired individuals

We sought to understand the extent to which Casp2 is involved in the production of Δtau314 proteins in the CI individuals. At present, there is no reliable method for measuring Casp2 activity in human brain tissue. Therefore, we measured levels of Casp2 detected by a monoclonal anti-Casp2 antibody directed against a C-terminal epitope (Table 1), which were previously shown to be elevated in Lewy body dementia^24^, Huntington’s disease^25^, and AD^26^.

**Figure 7:**
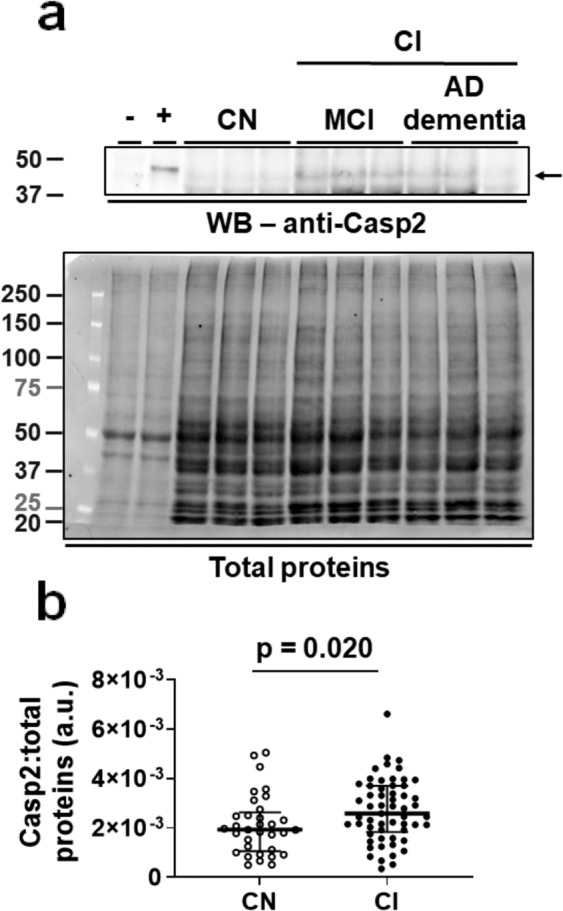
Levels of Casp2 are higher in the cognitively impaired individuals. (**a**) Representative blots showing that Casp2 (~48-kDa full-length form (arrow), upper panel) and total proteins (lower panel) were detected. The full-length Casp2 blot is presented in Supplementary Fig. S9, and there was no grouping within that blot. -, *Casp2* KO mice (negative control); +, C57BL/6J mice (positive control). CI, cognitively impaired individuals; CN, cognitively normal individuals; AD dementia, patients with Alzheimer’s disease dementia; MCI, individuals with mild cognitive impairment; a.u. = arbitrary unit. (**b**) Comparison of levels of Casp2 in the CI (*n* = 57) versus the CN individuals (*n* = 33). Mann-Whitney test was used for between-group comparison; medians (middle long bars) and 1^st^ (lower short bars) and 3^rd^ (upper short bars) quartiles are shown.

Levels of Casp2 proteins, normalized to levels of total proteins, were 1.3-fold higher in the CI individuals than in the CN individuals (Fig. 7, Supplementary Fig. S9, Table 3) and trended higher following adjustments for age at death, sex, and PMI of brain tissue harvest (Table 3). This finding supports the active involvement of Casp2 in Δtau314 production in persons affected with AD.

## Discussion

In this study, we detected and measured the levels of Δtau314 proteins, a C-terminally-truncated, soluble tau species whose production is responsible for mouse memory impairment and synaptic dysfunction^16^ in the ITG, one of the earliest brain regions affected by AD pathology^27^. In addition, we showed that Δtau314 proteins are derived from all six tau splicing isoforms, that levels of both Casp2 and Δtau314 proteins are higher in cognitively impaired than in normal individuals, and that levels of Δtau314 proteins are moderately predictive of cognitive impairment.

Our results support the findings of a previous study^16^, in which similar experimental procedures and quantitative biochemical analyses were utilized, and levels of Δtau314 proteins were measured in the same brain structure (*i.e*., ITG). Specifically, in the Zhao *et al*. study, levels of Δtau314 proteins in the CI individuals are 1.4-fold higher than in the CN individuals (Supplementary Fig. S10), and levels of T-tau proteins are 1.5-fold lower in the CI individuals than in the CN individuals (Supplementary Fig. S10), resulting in a 2.0-fold higher Δtau314:T-tau ratio in the CI individuals than in the CN individuals (Supplementary Fig. S10). However, unlike the Zhao *et al*. study, we did not normalize levels of Δtau314 and tau proteins to levels of βIII-tubulin because levels of Δtau314 proteins correlated inversely with levels of βIII-tubulin (Supplementary Fig. S11).

When measured by IP/WB, the levels of soluble T-tau proteins in the CI individuals were lower by approximately 20% than in the CN individuals, and yet quantification of βIII-tubulin levels indicates no obvious difference in the degree of neuronal loss between the two groups. This may be related to a higher degree of neurofibrillary tau pathology in the CI individuals than in the CN individuals (Supplementary Fig. S3b). It is reported that higher severity of neurofibrillary tau pathology associate with lower soluble tau levels in cortices of patients with AD dementia, but such an association is not obvious in age-matched controls^28^. While neuronal loss and age-related decrease in tau production may lower soluble tau levels in elderly CN individuals, a further loss of soluble tau proteins may be linked to the presence of neurofibrillary tau pathology in the studied cortical region of CI individuals. This is likely accomplished, at least partially, through reallocation of tau proteins from the soluble to the insoluble pool^28^.

The levels of soluble T-tau proteins, to which levels of Δtau314 proteins are normalized, show discrepancy between IP/WB and simoa. In our opinion, this is largely due to difference in experimental settings. Specifically, levels of full-length tau proteins were measured in direct WB; however, the use of an antibody targeting a mid-region tau epitope as the capture reagent in simoa likely results in measuring additional C-terminally truncated tau species besides the full-length proteins. To minimize discrepant results between IP/WB and simoa, the two assays should be set up to allow measuring virtually, if not exactly, the same proteins and their variants, and it is reasonable to use the same set of capture and detection reagents in IP/WB and simoa for quantification of proteins of interest, though the accessibility of antibodies to their targeted epitopes may differ under the denaturing WB versus the non-denaturing simoa detection conditions (see below for more discussion).

Although full-length tau isoforms are generally considered highly soluble proteins free of structure, recently solved atomic resolution structures of tau microtubule-binding domain in complex with cytoskeleton proteins^29,30^ suggest that tau adopts particular tertiary structures upon interacting with its binding partners in biological systems. Under non-denaturing experimental conditions like simoa and immunoprecipitation, it is conceivable that certain tau epitopes targeted by antibodies were inaccessible to the capture/detection reagents, resulting in inaccurate quantification and discrepant outcomes between different types of assays. Despite this, the consistent findings of Δtau314 proteins between IP/WB and simoa using 4F3 under different schemes (the capture reagent of simoa under non-denaturing conditions versus the detection reagent of IP/WB under denaturing conditions) suggest the issue of epitope mask does not apply to this Δtau314-specific monoclonal antibody. However, it may affect the measurement of soluble T-tau. To minimize the inaccuracy and inconsistency of immunoassay, protein quantities may need to be more comprehensively determined using available reagents that target different regions of proteins of interest, and assays may need to be carried out under light denaturing conditions to rid or attenuate the influence of epitope mask.

It is well established that tau proteins are digested by multiple proteases to produce various types of cleavage products (for reviews, see, for example^5^). A growing body of evidence supports the presence of truncated tau species produced in brains of individuals with dementia and their relevance to cognitive dysfunction^31–37^. Further, recent studies addressed the contribution of protease-mediated tau cleavage to synaptic dysfunction and animal behavioral abnormalities. There are other pathogenic pathways triggered by the proteolysis of tau besides Casp2-mediated tau cleavage at D314^16^. For example, Zhang *et al*. showed that asparagine endopeptidase (AEP)-mediated tau cleavage at asparagines 255 and 368 is responsible for synaptic dysfunction and cognitive deficits of tau transgenic mice^38^ modeling frontotemporal dementia with parkinsonism^39–41^, and that the tau cleavage product ending C-terminally at asparagine 368 is present in AD brains^42^. Indeed, our IP/MS analysis of tau proteins extracted from brain tissue identified both AEP-mediated cleavage products (Supplementary Fig. S12). Interestingly, besides peptides ending at the C-termini of Δtau314 species and AEP-cleaved tau products, using IP/MS we did not detect any other tau fragments that match the C-termini of truncated products (e.g., Δtau421^43^, tauΔCsp-6^44^, and C-terminally truncated tau at glutamate 391^45^) comprised of paired helical filaments of tau pathology in human brains. This failure is possibly due to the scarcity of these aggregation-prone tau proteins in the prepared brain homogenates.

Of particular note, we need to bear in mind the following points when interpreting the results. First, that the levels of both Casp-2 and Δtau314 proteins in the CI individuals are higher than in the CN individuals and that levels of Δtau314 proteins moderately predict cognitive impairment are correlative findings. Therefore, these results do *not* necessarily mean that Casp-2 or Δtau314 proteins play a causative role in deteriorating cognition. Rather, they suggest that Casp-2 and Δtau314 proteins are potentially involved in molecular pathways that lead to cognitive impairment. Interestingly, elevated levels of Casp-2 and Δtau314 proteins have been reported in multiple neurodegenerative disorders other than AD dementia^24–26^, thus indicating that Casp-2 and Δtau314 proteins may play a role in distinct disease processes. What remains unclear is whether the elevation of Casp-2 and Δtau314 proteins initiates or is secondary to neuropathologies and/or disease symptoms. Second, although beyond the scope of this study, the disease relevance of the finding that Casp-2-mediated Δtau314 production triggers cognitive impairment needs to be further validated. The identification of the presence of Δtau314 proteins and the role of Casp-2-mediated Δtau314 production in affecting cognition was carried out initially using mouse models that overexpress human tau transgene, a circumstance distinct from pathophysiological conditions of neurodegenerative disorders. Although we have subsequently confirmed the presence of Δtau314 proteins in the brain of individuals with varied tauopathies^16,24,25^, future studies are required to validate the cognition-affecting role of Casp-2-mediated Δtau314 production in systems that better model the diseases (*e.g*., human tau knock-in mice). Third, the possible physiological function of Casp-2-mediated Δtau314 production warrants an investigation. A growing body of evidence indicates that post-translationally modified tau (*e.g*., phosphorylated tau) is essential to neuronal development (*e.g*., neurite polarity and axon outgrowth) and normal cellular activities (*e.g*., axonal transport and synaptic plasticity) (for reviews, see for example^46,47^). While Casp-2-mediated Δtau314 production impairs cognition in tau overexpression models, and Δtau314 proteins are associated with CI individuals with long-standing pathology, that Casp-2-mediated Δtau314 production exerts physiological effects cannot be ruled out.

We were aware of a low sample size (*n* = 55 for the CI group and *n* = 30 for the CN group) used in the Zhao *et al*. study^16^; unfortunately, we have maximized the number of available individuals that met the criteria for specimen selection in that cohort. We, therefore, took an alternative strategy by conducting a similar investigation on a different cohort, hence this study. We admitted that conclusions of this study were drawn from a small population as well; however, the consistent findings from both cohorts increase the likelihood that our findings hold true in a larger population. In addition, we are currently extending the study by investigating the connection between levels of Δtau314 proteins in ITG and various functional and structural biomarkers derived from longitudinal studies of CN elderly individuals. Results of this ongoing study, with a sample size of nearly one hundred, would help us better understand the correlation of brain levels of Δtau314 proteins to the degree of progression in pathology and the rate of deterioration in cognitive function.

Given the minute portion of the brain in which Δtau314 measurement was made, the moderate associations between Δtau314 and clinical status is important. One limit of this study is that levels of Δtau314 proteins were only measured in inferior temporal gyrus. As the distribution of tau pathology varies dramatically among brain regions^48^, quantitative analysis of Δtau314 proteins in other (AD dementia-affected) regions, as one of our future study subjects, may help us better understand the connection between this tau fragment and cognition. In addition, simoa for quantification of Δtau314 proteins in lumbar puncture cerebrospinal fluid (CSF) and blood is currently under development. Successful measurement of Δtau314 protein levels in biological fluids of living persons allows their quantities to be tracked longitudinally and their predictive value for cognitive impairment to be better assessed. Further, the possibility of using Δtau314 proteins, alone or together with other established biomarkers, for dementia disease diagnosis can be investigated.

## Conclusions

Overall, our results confirm—in a second cohort—the presence of Δtau314 proteins in human brains, and the finding that levels of Δtau314 species are elevated in elderly CI individuals. In parallel, we showed elevated levels of Casp2 in CI individuals. We also validated 4F3, a novel Δtau314-specific monoclonal antibody, and we developed a highly sensitive ELISA for measuring Δtau314 levels using this antibody. We showed that Δtau314 proteins were generated from all six tau splicing isoforms. Finally, within the limits of our MS detection method, there appear to be only two classes of N-terminally intact, C-terminally truncated soluble tau species in the brain—those cleaved by Casp2 and AEP. These findings add to our knowledge the composition of Δtau314 proteins and reveal the relationships between Δtau314 proteins and cognition and neuropathology of individuals with AD pathology. Moving forward, the following subjects warrant further investigation: the topographic distribution of Δtau314 proteins in various anatomical brain regions of individuals at different stages of AD, the relationships of Δtau314 proteins to AD risk factors (*e.g*., aging and Apolipoprotein E genotype), the detection and quantitative analysis of Δtau314 proteins in biological fluids (*e.g*., lumbar puncture CSF and blood), and the correlation between catalytically active Casp2 and Δtau314. These studies would advance our understanding of the contribution of Casp2-mediated tau cleavage at D314 to disease pathogenesis and the potential use of Δtau314 proteins as molecular diagnostic markers of synaptic dysfunction.

## Methods

### *In vitro* synthesis of human tau proteins

Human tau proteins was synthesized *in vitro* as previously described^25^. Briefly, complementary DNA encoding *MAPT* (0N4R) was cloned into the pcDNA3.1^(+)^ vector (Thermo Fisher Scientific, Waltham, MA). For synthesis of the truncated Δtau421 protein, the trinucleotides encoding serine 422 were modified to a stop codon using the QuikChange II Site-Directed Mutagenesis Kit (Agilent Technologies, Santa Clara, CA) following the manufacturer’s instructions. The following primers were used for the mutagenesis: forward primer, 5′–AGCATCGACATGGTAGACTAGCCCCAGCTC–3′; reverse primer, 5′–GAGCTGGGGCTAGTCTACCATGTCGATGCT–3′. Similarly, site-directed mutagenesis was performed for synthesis of the truncated Δtau314 protein. The following primers were used for the mutagenesis: forward primer, 5′–AGTCTACAAACCAGTTGACTAGAGCAAGGTGACCTCCAAG–3′; reverse primer, 5′–CTTGGAGGTCACCTTGCTCTAGTCAACTGGTTTGTAGACT–3′. Tau protein syntheses were performed using a TnT Coupled Reticulocyte Lysate Systems (Promega, Madison, WI) following the manufacturer’s instructions.

### Human brain collection

Informed consent for brain donation was obtained from all participants and/or their legal guardians during enrollment. De-identified brain specimens of the ITG (Brodmann area 20) region were obtained from ninety elderly individuals at the Mayo Clinic, Rochester, MN. Frozen tissue was collected in polypropylene bags and stored at −80 °C prior to shipment to the University of Minnesota, Twin Cities, MN. All procedures were approved by the Institutional Review Boards (IRBs) of the Mayo Clinic and the University of Minnesota. All research was performed in accordance with relevant guidelines and regulations of the IRBs of the Mayo Clinic and the University of Minnesota.

### Mouse brain collection

C57BL/6J (Jackson Laboratory, Bar Harbor, ME; stock #000664) and *Casp2* knock-out (KO) (B6.129S4-*Casp2*^*tm1Yuan*^/J; Jackson Laboratory; stock #007899) mice were sacrificed at postnatal day 1, and brains were dissected immediately. All experiments involving mice were conducted in full accordance with the Association for Assessment and Accreditation of Laboratory Animal Care and the Institutional Animal Care and Use Committee (IACUC) guidelines at the University of Minnesota. The experimental protocol (ID: 1801-35505A) was approved by the IACUC at the University of Minnesota (effective Mar 12, 2018 through Mar 11, 2021).

### Brain protein extraction

For the detection and quantitative analysis of Δtau314 proteins, soluble total tau (T-tau) proteins, and βIII-tubulin, a two-step fractionation protocol adapted from a previously published experimental procedure^49^ was used to prepare intracellularly-enriched (IC) protein extracts. Briefly, ~300 mg (wet weight) of brain tissue was mechanically homogenized in 500 μL of extraction buffer 1 (50 mM tris(hydroxymethyl)aminomethane-hydrochloric acid (Tris-HCl, pH 7.6), 150 mM NaCl, 2 mM ethylenediaminetetraacetic acid (EDTA), 0.1% (weight/volume (w/v)) sodium dodecyl sulfate (SDS), and 0.01% (volume/volume (v/v)) Nonidet P-40 (NP-40) with the following protease and phosphatase inhibitors: 0.1 mM phenylmethylsulfonyl fluoride, 0.2 mM 1,10-phenanthroline monohydrate, protease inhibitor cocktail (MilliporeSigma, Burlington, MA) and phosphatase inhibitor cocktails (MilliporeSigma)). Supernatants were collected after centrifugation (800 *g*; 10 min, 4 °C) to obtain soluble, extracellularly-enriched proteins. The resulting pellets were homogenized in 500 μL of extraction buffer 2 (50 mM Tris-HCl (pH 7.6), 150 mM NaCl, and 0.1% (v/v) polyethylene glycol p-(1,1,3,3-tetramethylbutyl)-phenyl ether (Triton X-100) with the protease and phosphatase inhibitors mentioned above) followed by centrifugation (16,100 *g*; 90 min, 4 °C). Supernatants were collected to obtain IC proteins and subsequently depleted of endogenous immunoglobulin G (IgG) with Sepharose 4 Fast Flow Protein G beads (GE Healthcare, Piscataway, NJ; 50 µL of slurry per 500 µL of sample). Protein extracts were then stored at −20 °C until further use.

For the detection and quantitative analysis of Casp2, aqueous brain protein extracts were prepared based on a protocol previously published^50^. Specifically, human tissue specimens of ~150 mg (wet weight) were cut, transferred to 5 volumes (1 g per 5 mL) of ice-cold extraction buffer A (25 mM Tris-HCl (pH 7.4), 140 mM NaCl, and 3 mM KCl with the protease and phosphatase inhibitors mentioned above), and homogenized using a Dounce homogenizer. The resulting material was centrifuged for 90 min (16,100 *g*; 4 °C). The supernatant was subsequently depleted of endogenous IgG with Protein G Sepharose 4 Fast Flow beads (GE Healthcare) and stored at −20 °C until further use. As a control, brains of C57BL/6J and *Casp2* KO mice were homogenized using ice-cold extraction buffer B (50 mM Tris-HCl (pH 7.5), 150 mM NaCl, 1% (v/v) NP-40, and 0.5% (w/v) deoxycholate with the protease and phosphatase inhibitors mentioned above). The resulting material was centrifuged for 90 min (16,100 *g*; 4 °C). The supernatant was subsequently stored at −20 °C until further use.

Protein concentrations of brain extracts were determined using a bicinchoninic acid protein assay kit (Thermo Scientific, Rockford, IL) according to the manufacturer’s instructions.

### Immunoprecipitation (IP)

Immunoprecipitation was performed as described previously^25^. To detect and determine levels of Δtau314 proteins, 300 μg of brain proteins from IC extracts were added to immunoprecipitation dilution buffer (IPDB: 50 mM Tris-HCl (pH 7.4) and 150 mM NaCl, containing the protease and phosphatase inhibitors) to make the final volume 500 µL. The diluted protein extracts were incubated with 10 μg of the tau-13 antibody (BioLegend, San Diego, CA) or mouse IgG (negative control). The resulting samples were then incubated with Protein G Sepharose 4 Fast Flow resin (GE Healthcare) at 4 °C for 14–16 hr. Resins were then washed in IP buffer A (50 mM Tris-HCl (pH 7.4), 300 mM NaCl, 1 mM EDTA, and 0.1% (v/v) Triton X-100 with the protease and phosphatase inhibitors), followed by IP buffer B (50 mM Tris-HCl (pH 7.4), 150 mM NaCl, 1 mM EDTA, and 0.1% (v/v) Triton X-100 with the protease and phosphatase inhibitors). The immunoprecipitated proteins were then eluted at 95 °C using loading buffer (500 mM Tris-HCl (pH 8.0), 24% (v/v) glycerol, 8% (w/v) SDS, 0.01% (w/v) Coomassie brilliant blue, 0.1% (v/v) phenol red, and 710 mM β-mercaptoethanol). Eluates were fractionated on 10% Tris-HCl gels (Bio-Rad, Hercules, CA) and transferred onto 0.2-μm nitrocellulose membranes at 0.4 A (constant current), 4 °C for 4 hr.

To understand which tau isoforms produce Δtau314 proteins, 300 μg of brain proteins from IC extracts of representative CN individuals, individuals with MCI and AD dementia patients were immunoprecipitated with 4 µg of the custom-produced rabbit polyclonal antibody H1485 (New England Peptide, Gardner, MA) (or rabbit IgG as negative control) using the protocol described above.

### Western blotting (WB)

WB was performed according to a previously published protocol^51^.

To test the specificity of antibodies directed against Δtau314 proteins, 3 µL of the rabbit reticulocyte lysates following *in vitro* synthetic reactions were size-fractionated by SDS-polyacrylamide gel electrophoresis (PAGE) using Criterion 10–20% Tris-Tricine Precast gels (Bio-rad) and then electrophoretically transferred onto nitrocellulose membranes at a constant current of 0.4 A for 4 hr at 4 °C. Blots were probed with H1485 (New England Peptide; 1:2,000, final concentration = 350 ng/mL), the custom-produced mouse monoclonal antibody 4F3 (Precision Antibody, Columbia, MD; 1:2,000, final concentration = 500 ng/mL), or the mouse monoclonal antibody tau-13 (BioLegend; 1:30,000, final concentration = 17 ng/mL). Following 5 × 5-min washes in wash buffer (10 mM Tris-HCl (pH 7.4), 200 mM NaCl, and 0.1% (v/v) polyoxyethylene (20) sorbitan monolaurate (Tween 20)), membranes were incubated at room temperature with either the horseradish peroxidase (HRP)-conjugated anti-mouse IgG (Thermo Fisher Scientific; 1:200,000, final concentration = 5 ng/mL) or the HRP-conjugated anti-rabbit IgG (Thermo Fisher Scientific; 1:200,000, final concentration = 5 ng/mL) for 1 hr. The membranes were then washed again (5 × 5-min in wash buffer) prior to being developed.

To test the specificity of isoform-specific tau antibodies, 42 ng (7 ng per isoform) of the six purified recombinant tau isoforms (MilliporeSigma) were size-fractionated by SDS-PAGE using Criterion 10% Tris-HCl Precast gels (Bio-rad). Blots were probed with the mouse monoclonal antibody tau-5 (Thermo Fisher Scientific; 1:30,000, final concentration = 17 ng/mL), a mouse monoclonal anti-3R tau antibody (MilliporeSigma; 1:5,000; culture supernatant, final concentration not determined), or a mouse monoclonal anti-4R tau antibody (MilliporeSigma; 1:5,000; culture supernatant, final concentration not determined). Following 5 × 5-min washes in wash buffer, membranes were incubated at room temperature with the HRP-conjugated anti-mouse IgG (Thermo Fisher Scientific; 1:200,000, final concentration = 5 ng/mL) for 1 hr. The membranes were then washed again (5 × 5-min in wash buffer) prior to being developed.

To understand which tau isoforms produce Δtau314 proteins, blots were probed with the tau-5 and the anti- 3R and 4R tau antibodies as described above.

To measure levels of Δtau314 proteins, blots were probed with H1485 (1:2,500, final concentration = 280 ng/mL) or the biotin-conjugated 4F3 (1:45,000, final concentration = 36 ng/mL). Following 5 × 5-min washes in wash buffer, membranes were incubated at room temperature with either the HRP-conjugated NeutrAvidin (Thermo Fisher Scientific; 1:5,000, final concentration = 200 ng/mL) for 10 min for blots probed with the biotin-conjugated 4F3, or the HRP-conjugated anti-rabbit IgG for 1 hr for blots probed with H1485. The membranes were then washed again (5 × 5-min in wash buffer) prior to being developed.

To measure levels of T-tau proteins, 10 μg of brain proteins from IC extracts were electrophoretically separated by SDS-PAGE using Criterion 10% Tris-HCl Precast gels (Bio-Rad) and then transferred onto nitrocellulose membranes. Blots were probed with tau-13 (MilliporeSigma; 1:30,000, final concentration not determined). Following 5 × 5-min washes in wash buffer, membranes were incubated at room temperature with the HRP-conjugated anti-mouse IgG (Thermo Fisher Scientific; 1:200,000, final concentration = 5 ng/mL) for 1 hr. The membranes were then washed again (5 × 5-min in wash buffer) prior to being developed.

To measure levels of βIII-tubulin, a protein almost exclusively expressed in neurons, 10 μg of brain proteins from IC extracts were electrophoretically separated by SDS-PAGE using Criterion 10% Tris-HCl Precast gels (Bio-Rad) and then transferred onto nitrocellulose membranes. Blots were probed with the monoclonal anti-βIII-tubulin antibody (MilliporeSigma; 1:200,000, final concentration = 5 ng/mL). Following 5 × 5-min washes in wash buffer, membranes were incubated at room temperature with the HRP-conjugated anti-mouse IgG (Thermo Fisher Scientific; 1:200,000, final concentration = 5 ng/mL) for 1 hr. The membranes were then washed again (5 × 5-min in wash buffer) prior to being developed.

To measure levels of Casp2, 100 μg of proteins from the aqueous brain extracts were electrophoretically separated by SDS-PAGE using 7.5% Criterion TGX stain-free protein gels (Bio-Rad) and then transferred onto nitrocellulose membranes. Fifty µg of proteins from brain homogenates of WT and *Casp2* KO mice (Jackson Laboratory) served as the positive and negative control, respectively. Blots were probed with the rabbit monoclonal anti-Casp2 antibody (Abcam, Cambridge, MA; 1:5,000, final concentration = 496 ng/mL). Following 5 × 5-min washes in wash buffer, membranes were incubated at room temperature with the HRP-conjugated anti-rabbit IgG (Thermo Fisher Scientific; 1:200,000, final concentration = 5 ng/mL) for 1 hr. The membranes were then washed again (5 × 5-min in wash buffer) prior to being developed.

In addition, total protein levels were used as a loading control for normalization of levels of Casp2. Proteins transferred to nitrocellulose membranes were revealed following the manufacturer’s instruction before blots were probed with anti-Casp2.

Western blots were developed using the West Pico electrochemiluminescence (ECL) detection system (Thermo Fisher Scientific), except for the blots probed with biotin-conjugated 4F3, which were developed using the West Femto detection system (Thermo Fisher Scientific). The blots probed with anti-Casp2 were developed using the Clarity Max Western ECL detection system (Bio-Rad). The Kodak Scientific Imaging film X-OMAT Blue XB (PerkinElmer, Bostin, MA) was used to reveal the blots except for total proteins and Casp2, which were revealed using Image Lab 6.0 (Bio-Rad). For each blot, a series of exposures, ranging from 1 sec to 5 min, was used to ensure that bands of interest fell within the linear range of detection. Signal intensities were quantified densitometrically using Optiquant (Packard Cyclone, Perkin-Elmer Life Sciences Inc., Boston, MA) except for levels of total proteins and Casp2, which were quantified using Image Lab 6.0.

Experimenters performing (IP/)WB were blind to the demographic and clinical features of participants. The mean levels of Δtau314 proteins and βIII-tubulin were determined from two independent experiments, and the mean levels of T-tau proteins were determined from three independent experiments. The mean protein levels were used for statistical analysis.

### Antibody immobilization on protein G magnetic beads

The tau-13 antibody was immobilized as previously described^52^. Briefly, the antibody was covalently linked to Protein G magnetic beads (Thermo Fisher Scientific; 200 µg of antibody per 1 mL of bead slurry) using dimethyl pimelimidate (Thermo Fisher Scientific) as the crosslinker. Prior to use, beads were pre-treated with IP buffers A and B to wash off any antibody not covalently linked to beads. To monitor any shedding of antibody from the beads, the beads were treated in elution buffer (100 mM glycine-HCl (pH 2.8) and 1% (w/v) *n*-Octyl *β*-D-thioglucopyranoside (OTG)). The eluate was collected and analyzed by WB using HRP-conjugated goat-anti-mouse IgG (Jackson ImmunoResearch Laboratories Inc., West Grove, PA).

### Preparation of samples for mass spectrometry

IC protein extracts (0.9 mg) of a randomly selected specimen were incubated overnight at 4 °C with tau-13-bound Protein G-coupled magnetic beads. Beads were then washed twice with wash buffer 1 (0.1% (w/v) OTG in IPDB (pH 7.4)) and subsequently twice with wash buffer 2 (1% (w/v) OTG in IPDB (pH 7.4)). Proteins were eluted using 30 µL of elution buffer at room temperature by agitation for 5 min. Elution was carried out three times, and the eluates were mixed and allocated into three parts, which accounted for 10%, 45%, and 45% of the total volume, respectively. These allocated eluates were size-fractionated on a Criterion 10% Tris-HCl Precast gel (Bio-Rad) and then cut into three pieces. The piece of gel containing 10% of the eluates was subjected to WB using H1485 as the detection antibody, during which time a piece of gel containing 45% of the eluates lanes was stored in a moist chamber at 4 °C, whereas the other piece was subjected to silver stain using a SilverXpress silver staining kit (Thermo Fisher Scientific) following the manufacturer’s instruction. After WB, the untreated gel pieces were overlaid on the film record using the molecular weight standards for alignment, and the pieces of unstained gel overlaying the bands of interest (migrating at ~45, ~40, ~35, and ~30 kDa) were excised. In parallel, the bands of interest (migrating at ~45, ~40, ~35, and ~30 kDa) shown in the gel piece with silver stain were excised as well.

### Mass spectrometry

#### In-gel trypsin digestion

In-gel trypsin digestion was performed according to a previously published procedure^53^. Briefly, gel pieces were cut into a size of approximately 2 mm^2^ × 1 mm (thickness of the gel), washed with addition of wash solution (50 mM ammonium bicarbonate (pH 7.8) and 50% (v/v) acetonitrile) at room temperature for 15 min, and then aspirated. The washing and aspiration steps were repeated once. The gel pieces were then incubated for dehydration with 100% (v/v) acetonitrile at room temperature for 1 min. The acetonitrile was aspirated, and buffer 1 (50 mM ammonium bicarbonate (pH 7.8) and 10 mM dithiothreitol) was added to the gel pieces and incubated at 56 °C for 1 hr. Buffer 1 was then aspirated, and 55 mM iodoacetamide was added and incubated with gel pieces at room temperature in the dark for 30 min to alkylate cysteine residues. The solution was then aspirated, and the gel pieces were washed twice at room temperature with wash solution. After washing, 100% (v/v) acetonitrile was incubated with the gel pieces at room temperature for 1 min and then aspirated. Trypsin solution was made (5 ng/µL sequencing grade trypsin (Promega) in buffer 2 (50 mM ammonium bicarbonate (pH 7.8) and 5 mM calcium chloride)) and added to each sample to cover the gel pieces. The samples were incubated on ice for 10 min, after which any non-absorbed trypsin solution was aspirated. Buffer 2 was added to cover the gel pieces. Samples were placed in a warm air incubator at 37 °C for 16 hr. The solution from each sample was transferred to a new 1.5 mL microcentrifuge tube, and 50% (v/v) acetonitrile/0.3% (v/v) formic acid was added to each sample for further extraction of peptides from gel pieces. The same sample extracts were pooled, and the process was repeated with 75% (v/v) acetonitrile/0.3% (v/v) formic acid. The pooled digests were frozen at −80 °C and dried in a speed vac. The resolubilized peptide mixtures were desalted using the Stage Tip technique with 3 M Empore styrenedivinylbenzene extraction disks according to a previously published protocol^54^. The final peptide mixtures were dried *in vacuo*.

### Liquid chromatography (LC) and mass spectrometry (MS)

Liquid chromatography (LC) and mass spectrometry (MS) were performed as previously described^55^.

All peptide separations were carried out using an Easy-nLC 1000 HPLC (Thermo Fisher Scientific). Samples (~200 ng) were loaded directly onto a 30 cm × 100-µm internal diameter fused silica PicoTip Emitter (New Objective, Woburn, MA) packed in-house with ReproSil-Pur C18-AQ (1.9 µm particle, 120 Å pore; Dr. Maish GmbH Ammerbuch, Germany) at a flow rate of 1 µL/min with aqueous solution containing 0.1% (v/v) formic acid and 2% (v/v) acetonitrile. Peptide elution was performed using a gradient of 5–7% solution B (A: 0.1% (v/v) formic acid in water and B: 0.1% (v/v) formic acid in acetonitrile) for 1 min, 7–35% solution B for 1 hr, and 35–60% solution B over 5 min at a flow rate of 200 µL/min.

The column was mounted in a nanospray source directly in line with an Orbitrap Fusion mass spectrometer (Thermo Fisher Scientific). Spray voltage was set at 2.1 kV in positive mode. The heated capillary was maintained at 275 °C. The acquisition method combined two scan events (*i.e*., a full scan and a parallel reaction monitoring (PRM) event) that target the doubly- and triply- charged precursor ions of the HVPGGGSVQIVYKPVD and VQIVYKPVD peptides without scheduling. The full scan event employed a mass-to-charge ratio (*m/z*) 380–1,500 mass selection, an orbitrap resolution of 120,000 (at *m/z* 200), a target automatic gain control (AGC) value of 200,000, and maximum fill times of 100 msec. The PRM event used an orbitrap resolution of 30,000 (at *m/z* 200), a target AGC value of 200,000, and maximum injection times 55 msec. The precursor ion generated from each targeted peptide was isolated using an isolation window of 1.6-*m/z*. Fragmentation was performed with a higher-energy collisional dissociation collision energy of 30%. MS/MS scans were collected using a scan range from 100–1,000 *m/z*. PRM data were collected in centroid mode.

### Mass spectral database search

Peaks Studio^56^ 8.5 (Bioinformatics Solutions, Inc, Waterloo, Ontario, Canada) was used for interpretation of MS/MS (mass spectra) and protein inference. Search parameters were: human (taxonomy ID 9606) protein sequence database from UniProt (http://www.uniprot.org/) downloaded on December 13, 2016 concatenated with the common lab contaminant database from http://www.thegpm.org/crap/; precursor mass error tolerance: 50.0 parts per million (ppm); fragment mass error tolerance: 0.1 Dalton; precursor mass search type: monoisotopic; no enzyme specificity; variable modifications: methionine oxidation and di-oxidation, cysteine carbamidomethylation, pyroglutamic acid, and protein N-terminal acetylation; maximum variable modifications per peptide: 2; false discovery rate calculation: On; spectra merge: Off; no charge state correction; and spectral filter quality: >0.65.

### Mass spectral data interpretation

Support for the detection of peptides from each supporting MS/MS spectrum was based on: 1) a minimum of five consecutive b- or y-type peptide fragment ions, 2) 1% peptide and protein false discovery rate threshold, and 3) precursor mass accuracy <7 ppm.

### Mass spectral data presentation

Extracted ion chromatogram (XIC) precursor peptide profiles for MS identification of Δtau314 proteins were plotted in MZmine 2.29^56^, and XIC fragment ion profiles for MS identification of Δtau314 proteins were generated from Skyline 4.1 viewer^57^.

### Enzyme-linked immunosorbent assay (ELISA)

Samples were analyzed using Quanterix ultra-sensitive simoa technology with the HD-1 analyzer (Quanterix, Lexington, MA).

Quantitative measurements of Δtau314 proteins were performed using 4F3-coated magnetic beads for protein capture and a combination of biotin-conjugated BT2/HT7 antibodies for detection. For the quantification assay, serially diluted recombinant human Δtau314 (tau 0N4R derivative) protein samples were used to generate the calibration curve. The lower limit of detection (LLOD), calculated as 2.5 standard deviations above the mean of background signal, was approximately 0.1 pg/mL. The lower limit of quantification (LLOQ) was determined to be approximately 1 pg/mL. The quantification of Δtau314 protein levels of all the studied individuals were carried out within the linear range of the calibration curve.

Soluble T-tau proteins were quantitatively measured using the Simoa Tau 2.0 reagent kit (Quanterix), which has been widely used in multiple published studies (see, for example^58–60^). The LLOD and LLOQ are reported as 0.019 and 0.061 pg/mL, respectively. Serially diluted recombinant full-length human tau (0N4R splicing isoform) protein samples were used to generate the calibration curve. The quantification of soluble T-tau protein levels of all the studied individuals was carried out within the linear range of the calibration curve.

Experimenters performing ELISA were blind to the demographic and clinical features of participants. The experiments of quantitative determination of the levels of both Δtau314 and T-tau proteins were performed in duplicate. The mean protein levels were used for statistical analysis.

### Statistical analysis

Statistical analyses were performed using GraphPad Prism Version 7.04 (GraphPad Software, La Jolla, CA) and R Version 3.4.1 (R Foundation for Statistical Computing, Vienna, Austria). The demographic and neuropathological characteristics were compared among the AD dementia, MCI, and CN individuals using Kruskal-Wallis tests for continuous variables and chi-square tests for binary variables. When overall differences were detected between groups, *post hoc* analyses comparing each pair of groups were performed. The associations between demographic and neuropathological characteristics and levels of Δtau314 proteins (normalized to levels of T-tau proteins) were estimated using Spearman’s rank-order correlations for continuous variables and Mann-Whitney tests for binary variables. Finally, levels of Casp2, Δtau314, T-tau, βIII-tubulin, and total proteins were compared using Mann-Whitney tests between the CN and the CI individuals and between the AD dementia and the MCI, respectively. In addition, multiple linear regressions of the log-transformed protein levels were fit with adjustment for sex, age at death, and PMI of brain tissue harvest. The unadjusted version of this analysis (*i.e*., a two-tailed, unpaired *t*-test of the log-transformed protein levels) was also shown. *P* < 0.05 was considered statistically significant. No formal corrections were made for the testing of multiple outcomes. The area under a curve (AUC) values were compared using DeLong’s tests for correlated receiver operating characteristic (ROC) curves.

## Supplementary information

Supplementary information.

## Data Availability

All data generated or analyzed during this study are included in this published article and its Supplementary Information Files.

## References

[1] Drechsel DN, Hyman AA, Cobb MH, Kirschner MW (1992). Modulation of the dynamic instability of tubulin assembly by the microtubule-associated protein tau. Mol. Biol. Cell.

[2] Gustke N, Trinczek B, Biernat J, Mandelkow EM, Mandelkow E (1994). Domains of tau protein and interactions with microtubules. Biochem..

[3] Goedert M, Spillantini MG, Jakes R, Rutherford D, Crowther RA (1989). Multiple isoforms of human microtubule-associated protein tau: sequences and localization in neurofibrillary tangles of Alzheimer’s disease. Neuron.

[4] Andreadis A, Brown WM, Kosik KS (1992). Structure and novel exons of the human tau gene. Biochem..

[5] Martin L, Latypova X, Terro F (2011). Post-translational modifications of tau protein: implications for Alzheimer’s disease. Neurochem. Int..

[6] Arriagada PV, Growdon JH, Hedley-Whyte ET, Hyman BT (1992). Neurofibrillary tangles but not senile plaques parallel duration and severity of Alzheimer’s disease. Neurol..

[7] Berg L (1998). Clinicopathologic studies in cognitively healthy aging and Alzheimer’s disease: relation of histologic markers to dementia severity, age, sex, and apolipoprotein E genotype. Arch. Neurol..

[8] Bierer LM (1995). Neocortical neurofibrillary tangles correlate with dementia severity in Alzheimer’s disease. Arch. Neurol..

[9] Giannakopoulos P (2003). Tangle and neuron numbers, but not amyloid load, predict cognitive status in Alzheimer’s disease. Neurol..

[10] Guillozet AL, Weintraub S, Mash DC, Mesulam MM (2003). Neurofibrillary tangles, amyloid, and memory in aging and mild cognitive impairment. Arch. Neurol..

[11] Gomez-Isla T (1997). Neuronal loss correlates with but exceeds neurofibrillary tangles in Alzheimer’s disease. Ann. Neurol..

[12] Santacruz K (2005). Tau suppression in a neurodegenerative mouse model improves memory function. Sci..

[13] Sydow A (2011). Tau-induced defects in synaptic plasticity, learning, and memory are reversible in transgenic mice after switching off the toxic Tau mutant. J. Neurosci..

[14] Van der Jeugd A (2012). Cognitive defects are reversible in inducible mice expressing pro-aggregant full-length human Tau. Acta Neuropathol..

[15] Kuchibhotla KV (2014). Neurofibrillary tangle-bearing neurons are functionally integrated in cortical circuits *in vivo*. Proc. Natl Acad. Sci. USA.

[16] Zhao X (2016). Caspase-2 cleavage of tau reversibly impairs memory. Nat. Med..

[17] Ramsden M (2005). Age-dependent neurofibrillary tangle formation, neuron loss, and memory impairment in a mouse model of human tauopathy (P301L). J. Neurosci..

[18] Hutton M (1998). Association of missense and 5′-splice-site mutations in tau with the inherited dementia FTDP-17. Nat..

[19] Dumanchin C (1998). Segregation of a missense mutation in the microtubule-associated protein tau gene with familial frontotemporal dementia and parkinsonism. Hum. Mol. Genet..

[20] Bennett DA (2012). Overview and findings from the rush Memory and Aging Project. Curr. Alzheimer Res..

[21] Braak H, Braak E (1991). Neuropathological stageing of Alzheimer-related changes. Acta Neuropathol..

[22] Montine TJ (2012). National Institute on Aging-Alzheimer’s Association guidelines for the neuropathologic assessment of Alzheimer’s disease: a practical approach. Acta Neuropathol..

[23] Mirra SS (1991). The Consortium to Establish a Registry for Alzheimer’s Disease (CERAD). Part II. Standardization of the neuropathologic assessment of Alzheimer’s disease. Neurol..

[24] Smith BR (2019). A soluble tau fragment generated by caspase-2 is associated with dementia in Lewy body disease. Acta Neuropathol. Commun..

[25] Liu P (2019). A soluble truncated tau species related to cognitive dysfunction and caspase-2 is elevated in the brain of Huntington’s disease patients. Acta Neuropathol. Commun..

[26] Shimohama S, Tanino H, Fujimoto S (1999). Changes in caspase expression in Alzheimer’s disease: comparison with development and aging. Biochem. Biophys. Res. Commun..

[27] Convit A (2000). Atrophy of the medial occipitotemporal, inferior, and middle temporal gyri in non-demented elderly predict decline to Alzheimer’s disease. Neurobiol. Aging.

[28] Mukaetova-Ladinska EB, Harrington CR, Roth M, Wischik CM (1993). Biochemical and anatomical redistribution of tau protein in Alzheimer’s disease. Am. J. Pathol..

[29] Cabrales Fontela Y (2017). Multivalent cross-linking of actin filaments and microtubules through the microtubule-associated protein Tau. Nat. Commun..

[30] Kadavath H (2015). Folding of the Tau Protein on Microtubules. Angew. Chem. Int. Ed. Engl..

[31] Garcia-Sierra F, Wischik CM, Harrington CR, Luna-Munoz J, Mena R (2001). Accumulation of C-terminally truncated tau protein associated with vulnerability of the perforant pathway in early stages of neurofibrillary pathology in Alzheimer’s disease. J. Chem. Neuroanat..

[32] Rissman RA (2004). Caspase-cleavage of tau is an early event in Alzheimer disease tangle pathology. J. Clin. Invest..

[33] Albrecht S (2007). Activation of caspase-6 in aging and mild cognitive impairment. Am. J. Pathol..

[34] Basurto-Islas G (2008). Accumulation of aspartic acid421- and glutamic acid391-cleaved tau in neurofibrillary tangles correlates with progression in Alzheimer disease. J. Neuropathol. Exp. Neurol..

[35] Henriksen K (2013). An enzyme-generated fragment of tau measured in serum shows an inverse correlation to cognitive function. PLoS One.

[36] Ramcharitar J, Afonso VM, Albrecht S, Bennett DA, LeBlanc AC (2013). Caspase-6 activity predicts lower episodic memory ability in aged individuals. Neurobiol. Aging.

[37] Ramcharitar J (2013). Cerebrospinal fluid tau cleaved by caspase-6 reflects brain levels and cognition in aging and Alzheimer disease. J. Neuropathol. Exp. Neurol..

[38] Yoshiyama Y (2007). Synapse loss and microglial activation precede tangles in a P301S tauopathy mouse model. Neuron.

[39] Sperfeld AD (1999). FTDP-17: an early-onset phenotype with parkinsonism and epileptic seizures caused by a novel mutation. Ann. Neurol..

[40] Bugiani O (1999). Frontotemporal dementia and corticobasal degeneration in a family with a P301S mutation in tau. J. Neuropathol. Exp. Neurol..

[41] Lossos A (2003). Frontotemporal dementia and parkinsonism with the P301S tau gene mutation in a Jewish family. J. Neurol..

[42] Zhang Z (2014). Cleavage of tau by asparagine endopeptidase mediates the neurofibrillary pathology in Alzheimer’s disease. Nat. Med..

[43] Gamblin TC (2003). Caspase cleavage of tau: linking amyloid and neurofibrillary tangles in Alzheimer’s disease. Proc. Natl Acad. Sci. USA.

[44] Guo H (2004). Active caspase-6 and caspase-6-cleaved tau in neuropil threads, neuritic plaques, and neurofibrillary tangles of Alzheimer’s disease. Am. J. Pathol..

[45] Novak M, Kabat J, Wischik CM (1993). Molecular characterization of the minimal protease resistant tau unit of the Alzheimer’s disease paired helical filament. EMBO J..

[46] Wang Y, Mandelkow E (2016). Tau in physiology and pathology. Nat. Rev. Neurosci..

[47] Arendt T, Stieler JT, Holzer M (2016). Tau and tauopathies. Brain Res. Bull..

[48] Jack CR (2018). Longitudinal tau PET in ageing and Alzheimer’s disease. Brain.

[49] Sherman MA, Lesne SE (2011). Detecting abeta*56 oligomers in brain tissues. Methods Mol. Biol..

[50] Shankar GM (2008). Amyloid-beta protein dimers isolated directly from Alzheimer’s brains impair synaptic plasticity and memory. Nat. Med..

[51] Liu P (2011). Grape seed polyphenolic extract specifically decreases abeta*56 in the brains of Tg2576 mice. J. Alzheimers Dis..

[52] Grant MKO (2019). Human cerebrospinal fluid 6E10-immunoreactive protein species contain amyloid precursor protein fragments. PLoS One.

[53] Thu YM (2016). Slx5/Slx8 Promotes Replication Stress Tolerance by Facilitating Mitotic Progression. Cell Rep..

[54] Rappsilber J, Ishihama Y, Mann M (2003). Stop and go extraction tips for matrix-assisted laser desorption/ionization, nanoelectrospray, and LC/MS sample pretreatment in proteomics. Anal. Chem..

[55] Brian, B. F. T. *et al*. Unique-region phosphorylation targets LynA for rapid degradation, tuning its expression and signaling in myeloid cells. *Elife* **8**, 10.7554/eLife.46043 (2019). 10.7554/eLife.46043 PMC6660195 31282857

[56] Ma B (2003). PEAKS: powerful software for peptide de novo sequencing by tandem mass spectrometry. Rapid Commun. Mass. Spectrom..

[57] MacLean B (2010). Skyline: an open source document editor for creating and analyzing targeted proteomics experiments. Bioinforma..

[58] Pase MP (2019). Plasma total-tau as a biomarker of stroke risk in the community. Ann. Neurol..

[59] Fossati S (2019). Plasma tau complements CSF tau and P-tau in the diagnosis of Alzheimer’s disease. Alzheimers Dement..

[60] Muller S (2017). Tau plasma levels in subjective cognitive decline: Results from the DELCODE study. Sci. Rep..

