# Author Correction: A soluble truncated tau species related to cognitive dysfunction is elevated in the brain of cognitively impaired human individuals

**DOI:** 10.1038/s41598-020-65178-8

**Published:** 2020-05-12

**Authors:** Peng Liu, Benjamin R. Smith, Michelle L. Montonye, Lisa J. Kemper, Kailee Leinonen-Wright, Kathryn M. Nelson, LeeAnn Higgins, Candace R. Guerrero, Todd W. Markowski, Xiaohui Zhao, Ashley J. Petersen, David S. Knopman, Ronald C. Petersen, Karen H. Ashe

**Affiliations:** 1 Department of Neurology, University of Minnesota, Minneapolis, MN 55455 USA; 2 Department of Neuroscience, University of Minnesota, Minneapolis, MN 55455 USA; 3 N. Bud Grossman Center for Memory Research and Care, University of Minnesota, Minneapolis, MN 55455 USA; 4 Institute for Therapeutics Discovery and Development, University of Minnesota, Minneapolis, MN 55455 USA; 5 Department of Biochemistry, Molecular Biology, and Biophysics, University of Minnesota, St. Paul, MN 55108 USA; 6 Division of Biostatistics, University of Minnesota, Minneapolis, MN 55455 USA; 7 Department of Neurology, Mayo Clinic, Rochester, MN 55905 USA; 8 Geriatric Research, Education, and Clinical Centers, Veterans Affairs Medical Center, Minneapolis, MN 55417 USA

Correction to: *Scientific Reports* 10.1038/s41598-020-60777-x, published online 02 March 2020

The original version of this Article contained errors. The present address for Xiaohui Zhao was incorrectly given as “AstraZeneca, Gaithersburg, MD, 20878, USA” which has now been removed from the Article.

As a result, the Competing Interests statement now reads:

“Xiaohui Zhao is currently an employee of AstraZeneca and contributed to this research before joining AstraZeneca.”

These have now been corrected in the PDF and HTML versions of this Article.

