# Supplementary material for: A soluble truncated tau species related to cognitive dysfunction is elevated in the brain of cognitively impaired human individuals
Source: Sci Rep. 2020 Mar 2;10:3869. doi: 10.1038/s41598-020-60777-x (PMC7052165; doi:10.1038/s41598-020-60777-x)
Supplement: Supplementary file 1 — Supplementary information. [file 41598_2020_60777_MOESM1_ESM.docx]

Supporting information

**A soluble truncated tau species related to cognitive dysfunction is elevated in the brain of cognitively impaired human individuals**

Peng Liu, Benjamin R. Smith, Michelle L. Montonye, Lisa J. Kemper, Kailee Leinonen-Wright, Kathryn M. Nelson, LeeAnn Higgins, Candace R. Guerrero, Todd W. Markowski, Xiaohui Zhao, Ashley J. Petersen, David S. Knopman, Ronald C. Petersen, Karen H. Ashe

**
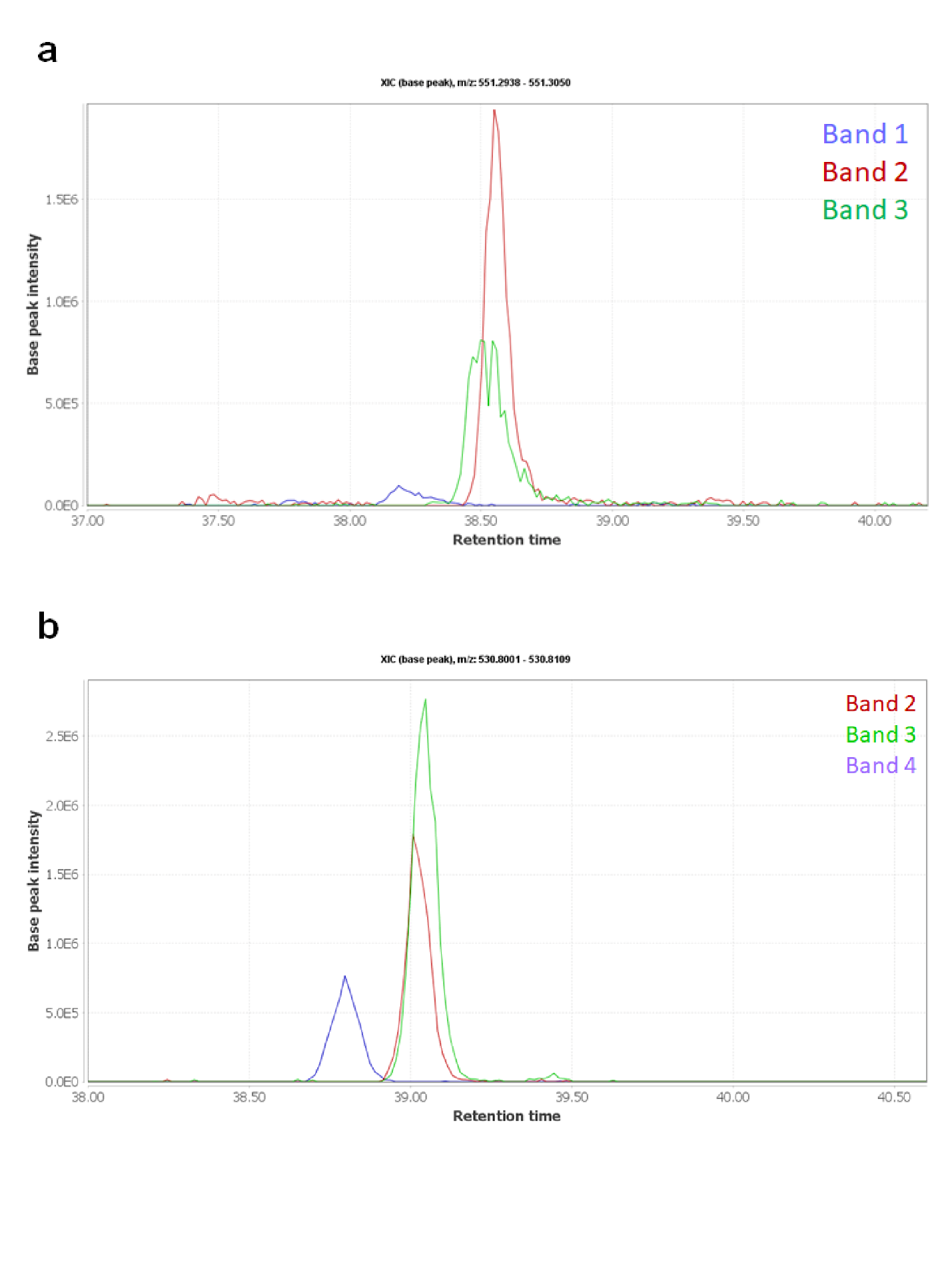
**

**Supplementary Figure S1 Extracted ion chromatogram (XIC) precursor peptide profiles for mass spectrometry identification of Δtau314 proteins.** (**a**) [M + 3H]^3+^ (theoretical monoisotopic *m/z* = 551.2994) for the peptide HVPGGGSVQIVYKPVD identified in bands 1-3. (**b**) [M + 2H]^2+^ (theoretical monoisotopic *m/z* = 530.8055) for the peptide VQIVYKPVD identified in bands 2-4. Chromatograms were plotted in MZmine 2.29^1^.


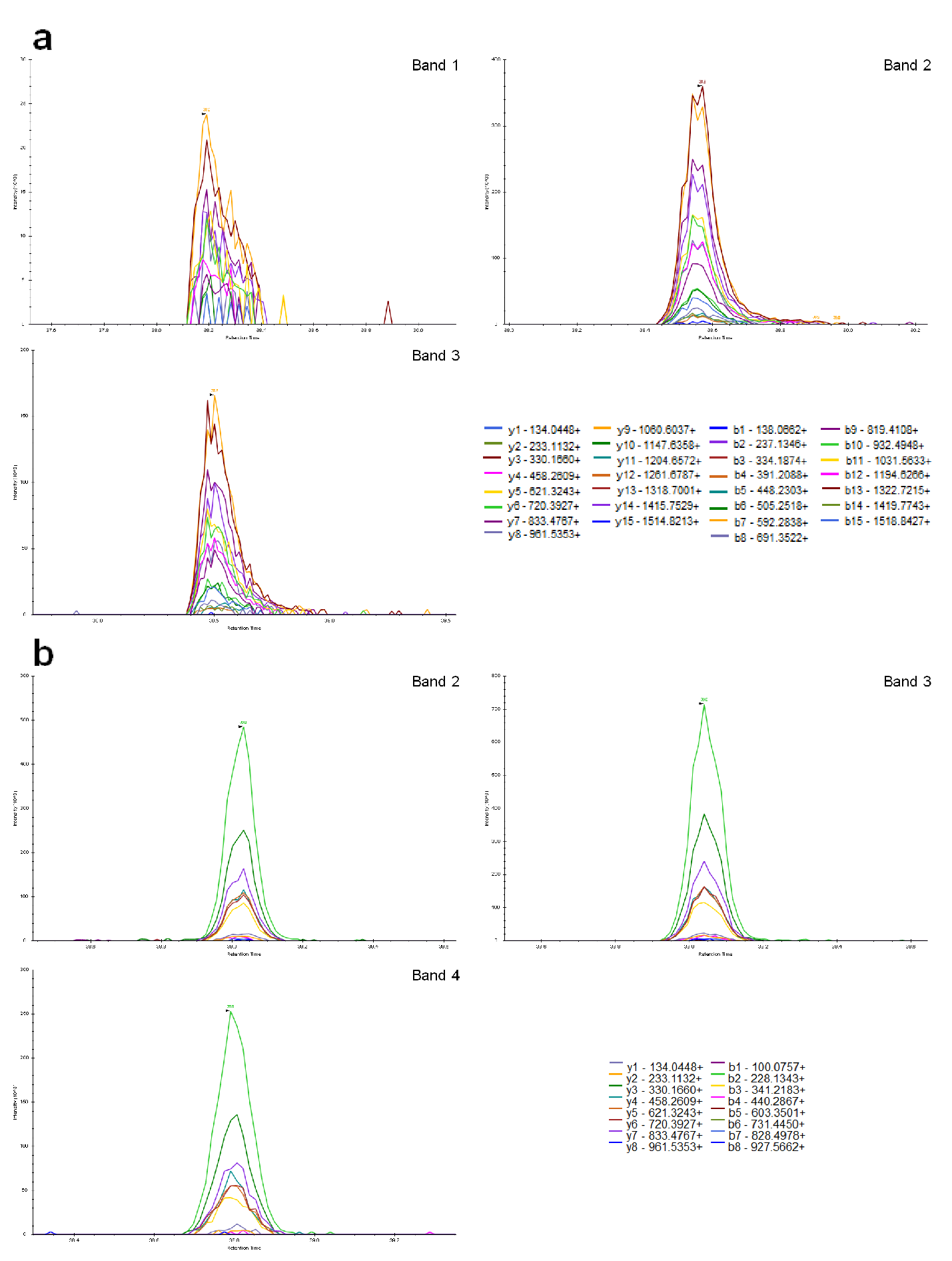


**Supplementary Figure S2 XIC fragment ion profiles for mass spectrometry identification of Δtau314 proteins.** (**a**) XIC fragment ion profiles from bands 1-3 for [M + 3H]^3+^ (theoretical monoisotopic *m/z* = 551.2994) for the peptide HVPGGGSVQIVYKPVD. (**b**) XIC fragment ion profiles from bands 2-4 for [M + 2H]^2+^ (theoretical monoisotopic *m/z* = 530.8055) for the peptide VQIVYKPVD. The identified XIC profiles are represented by the color legend for their respective b or y ions. The profiles were generated from Skyline 4.1 viewer^2^.





**Supplementary Figure S3 *Post hoc* comparison of the demographic and neuropathological characteristics of the human individuals with different diagnoses.** (**a**) The ages at death of the cognitively normal (CN) individuals are lower than the Alzheimer’s disease dementia (AD dementia) patients. The ages at death between the AD dementia patients and the individuals with mild cognitive impairment (MCI) do not differ, nor do the ages at death between the MCI and the CN individuals. (**b**) The Braak stages of the CN individuals are lower than the individuals with MCI and the AD dementia patients, and the Braak stages of the individuals with MCI are lower than the AD dementia patients. (**c**) The consortium to establish a registry for Alzheimer's disease (CERAD) scores for density of neocortical neuritic plaque of the CN individuals are lower than the individuals with MCI and the AD dementia patients. The CERAD neuritic plaque density scores between the individuals with MCI and the AD dementia patients do not differ. Dunn’s *post hoc* analyses were used following Kruskal-Wallis tests. Median (middle long bars) and 1^st^ (lower short bars) and 3^rd^ (upper short bars) quartiles are shown. Yr, years.





**Supplementary Figure S4 Levels of Δtau314 proteins do not differ between the AD dementia patients and the individuals with MCI.** (**a**, **b**) Comparison of levels of the H1485- (a) and the 4F3- reactive (b) proteins between the Alzheimer’s disease dementia (AD dementia) patients (*n* = 24) and the individuals with mild cognitive impairment (MCI, *n* = 33). (**c**) Comparison of levels of the tau-13-reactive soluble total tau (T-tau) proteins between the AD dementia patients and the individuals with MCI. (**d**, **e**) Comparison of levels of the H1485- (d) and the 4F3- reactive (e) proteins between the AD dementia patients and the individuals with MCI following normalization to the levels of the tau-13-reactive T-tau proteins. (**f**) Comparison of levels of the Δtau314 proteins determined using an ultra-sensitive ELISA between the AD dementia patients (*n* = 23, one was missing due to shortage of sample supply) and the individuals with MCI (*n* = 33). Notably, the y-axes in figures d-f are in the log scale. Mann-Whitney tests were used; medians (middle long bars) and 1^st^ (lower short bars) and 3^rd^ (upper short bars) quartiles are shown.





**Supplementary Figure S5 The full-length blot of Fig. 4c.** The bands spanning the vertical bar were shown in Fig. 4c.





**Supplementary Figure S6 The relationship of Δtau314 protein levels with CERAD scores for density of neocortical neuritic plaque of individuals.** (**a-b**) The correlations of levels of the H1485-reactive Δtau314 proteins normalized to soluble total tau (T-tau) protein levels (a) and levels of the 4F3-reactive Δtau314 proteins normalized to T-tau protein levels (b) to CERAD neuritic plaque density scores of subjects. The numbers of analyzed subjects in varied CERAD neuritic plaque density scores are shown in parentheses. Spearman’s rank-order correlation analyses were used. CN, cognitively normal individuals; MCI, individuals with mild cognitive impairment; AD dementia, patients with Alzheimer’s disease dementia.





**Supplementary Figure S7 The relationship of Δtau314 protein levels with Braak stages of individuals.** (**a-b**) The correlations of levels of the H1485-reactive Δtau314 proteins normalized to soluble total tau (T-tau) protein levels (a) and levels of the 4F3-reactive Δtau314 proteins normalized to T-tau protein levels (b) to Braak stages of subjects. The numbers of analyzed subjects in varied Braak stages are shown in parentheses. Spearman’s rank-order correlation analyses were used. CN, cognitively normal individuals; MCI, individuals with mild cognitive impairment; AD dementia, patients with Alzheimer’s disease dementia.





**Supplementary Figure S8 The relationships of Δtau314 protein levels with the demographic characteristics of participants.** (**a-b**) The correlations of levels of the H1485-reactive Δtau314 proteins normalized to soluble total tau (T-tau) protein levels (a) and levels of the 4F3- reactive Δtau314 proteins normalized to T-tau protein levels (b) to ages at death of individuals. (**c-d**) Levels of the H1485-reactive Δtau314 proteins normalized to T-tau protein levels (c) and levels of the 4F3- reactive Δtau314 proteins normalized to T-tau protein levels (d) in the female and the male individuals. The numbers of analyzed subjects are shown in parentheses. Notably, the y-axes are in the log scale. (**e-f**) The correlations of levels of the H1485-reactive Δtau314 proteins normalized to T-tau protein levels (e) and levels of the 4F3-reactive Δtau314 proteins normalized to T-tau protein levels (f) to post-mortem intervals of brain harvest. For figures a, b, e, and f, Spearman’s rank-order correlation analyses were used. For figures c and d, Mann-Whitney tests were used; median (middle long bars) and 1^st^ (lower short bars) and 3^rd^ (upper short bars) quartiles are shown. CN, cognitively normal individuals; MCI, individuals with mild cognitive impairment; AD dementia, patients with Alzheimer’s disease dementia. Yr, years.





**Supplementary Figure S9 The full-length blot of Fig. 7a, upper panel.** The bands highlighted by an arrow were shown in Fig. 7a, upper panel.





**Supplementary Figure S10 Comparison of levels of Δtau314 and tau proteins in participants of the Zhao *et al.* study.** (**a**) Comparison of levels of the tau-13-immunoprecipitated, H1485-reactive Δtau314 proteins between the cognitively normal (CN) individuals (*n* = 30) and the cognitively impaired (CI) individuals (*n* = 55). (**b**) Comparison of levels of the tau-13-reactive soluble total tau (T-tau) proteins between the CN and the CI individuals. (**c**) Comparison of levels of the tau-13-immunoprecipitated, H1485-reactive Δtau314 proteins, normalized to levels of T-tau proteins, between the CN and the CI individuals. Mann-Whitney tests were used; medians (middle long bars) and 1^st^ (lower short bars) and 3^rd^ (upper short bars) quartiles are shown. Notably, the y-axes in figure c is in the log scale.





**Supplementary Figure S11 Levels of Δtau314 proteins are correlated inversely with levels of βIII-tubulin.** Levels of the H1485- (**a**) and the 4F3- reactive (**b**) Δtau314 proteins determined by IP/WB inversely correlate to level of βIII-tubulin determined by WB. Spearman’s rank-order correlation analyses were used. Notably, the y-axes in figures are in the log scale. CN, cognitively normal individuals; MCI, individuals with mild cognitive impairment; AD dementia, patients with Alzheimer’s disease dementia.


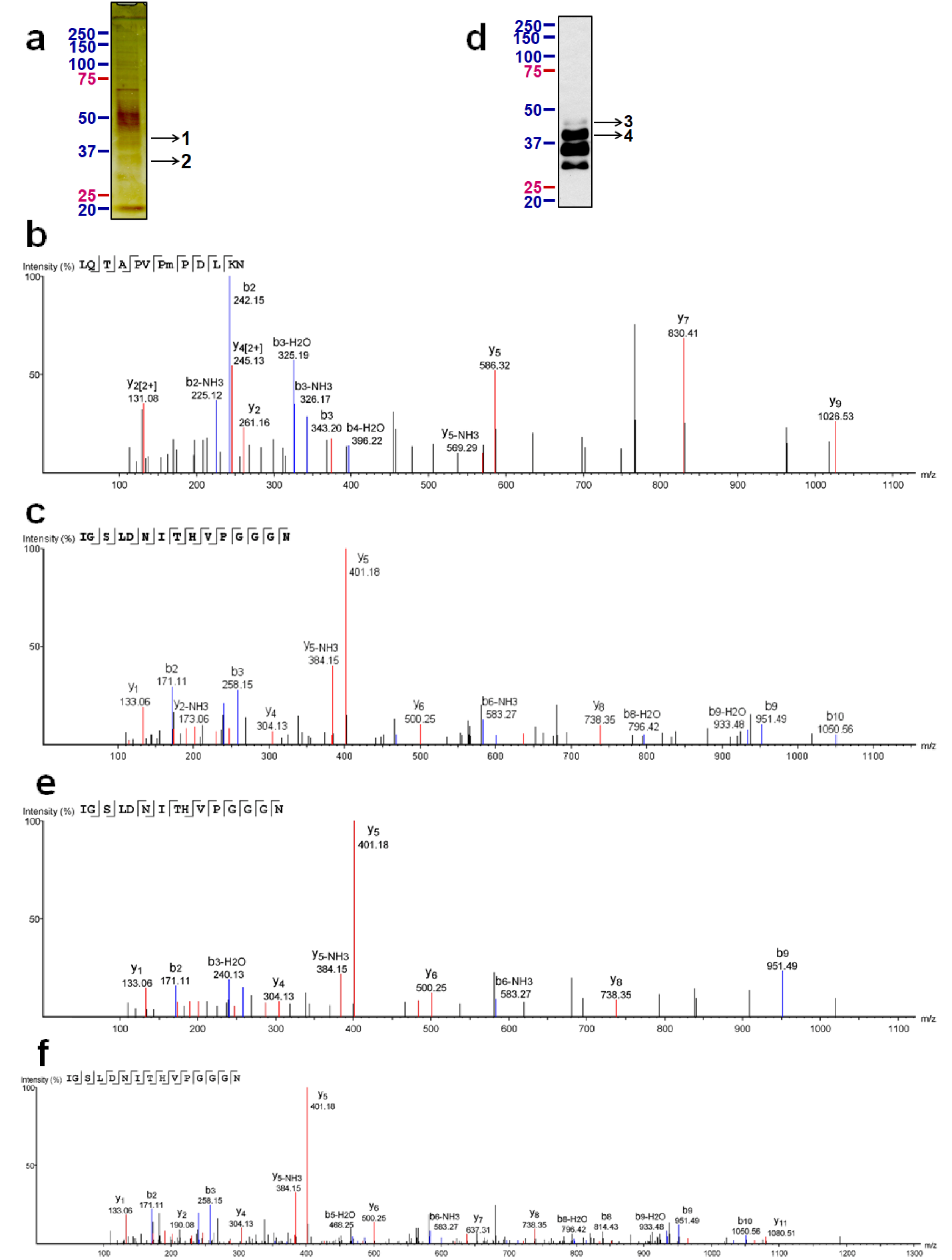


**Supplementary Figure S12 Asparagine endopeptidase (AEP)-mediated tau cleavage products in the inferior temporal gyrus are identified using immunoprecipitation coupled to mass spectrometry.** (**a**) A silver stain showing electrophoretically fractionated proteins of eluates of the tau-13-immunoprecipitated brain extracts. (**b,** **c**) Tandem mass spectrometry (MS/MS) spectra showing in bands 1 (c) and 2 (b) the identification of the peptides LQTAPVPmPDLKN (m = oxidized methionine) and IGSLDNITHVPGGGN, respectively. These two peptides correspond to the C-termini of the AEP-cleaved tau fragments at N255 and N368, respectively. (**d**) An H1485-probed Western blot of eluates of the tau-13-immunoprecipitated brain extracts. (**e,** **f**) MS/MS spectra showing in bands 3 (e) and 4 (f) the identification of the peptide IGSLDNITHVPGGGN, which corresponds to the C-terminus of the AEP-cleaved tau fragment at N368.

**Supplementary Table S1 Demographic and neuropathological characteristics of participants**

| **ID** | **Diagnosis^a^** | **Age (yr)^b^** | **Sex** | **PMI (hr)^c^** | **BST^d^** | **CERAD^e^** |
| --- | --- | --- | --- | --- | --- | --- |
| 1 | MCI | 87 | M | 37.1 | 4 | 2 |
| 2 | CN | 89 | F | 12.3 | 2 | 1 |
| 3 | CN | 91 | F | 12.8 | 3 | 0 |
| 4 | MCI | 87 | M | 3.2 | 2 | 3 |
| 5 | CN | 84 | F | 10.8 | 2 | 0 |
| 6 | MCI | 88 | F | 18.6 | 4 | 1 |
| 7 | CN | 81 | M | 8.9 | 2 | 0 |
| 8 | CN | 91 | F | 18.8 | 2 | 0 |
| 9 | AD dementia | 89 | M | 20 | 4 | 0 |
| 10 | AD dementia | 87 | M | 18 | 4 | 3 |
| 11 | MCI | 92 | F | 3.4 | 5 | 2 |
| 12 | MCI | 94 | F | 2.5 | 3 | 0 |
| 13 | CN | 82 | M | 18 | 2 | 1 |
| 14 | MCI | 84 | M | 2 | 5 | 2 |
| 15 | CN | 88 | M | 5 | 2 | 2 |
| 16 | AD dementia | 85 | F | 11.5 | 5 | 1 |
| 17 | MCI | 91 | M | 4 | 4 | 1 |
| 18 | CN | 82 | M | 3.8 | 2 | 0 |
| 19 | CN | 87 | F | 4 | 4 | 1 |
| 20 | MCI | 94 | F | 15.3 | 4 | 0 |
| 21 | AD dementia | 94 | F | 30 | 5 | 3 |
| 22 | CN | 77 | F | 4.2 | 3 | 0 |
| 23 | CN | 75 | F | 24.1 | 2 | 0 |
| 24 | AD dementia | 92 | M | 22.1 | 4 | 2 |
| 25 | AD dementia | 92 | F | 13 | 5 | 2 |
| 26 | MCI | 96 | F | 9.3 | 2 | 0 |
| 27 | CN | 93 | F | 16.4 | 1 | 0 |
| 28 | CN | 86 | M | 16.8 | 1 | 0 |
| 29 | CN | 93 | M | 13 | 3 | 2 |
| 30 | AD dementia | 93 | F | 2 | 4 | 0 |
| 31 | AD dementia | 90 | F | 11.5 | 5 | 2 |
| 32 | AD dementia | 99 | F | 9.5 | 5 | 1 |
| 33 | CN | 90 | M | 16.8 | 2 | 1 |
| 34 | CN | 77 | F | 19.8 | 2 | 1 |
| 35 | MCI | 86 | F | 34.6 | 4 | 2 |
| 36 | MCI | 89 | M | 10.5 | 4 | 2 |
| 37 | MCI | 93 | F | 19 | 2 | 1 |
| 38 | CN | 94 | F | 3.2 | 2 | 0 |
| 39 | CN | 90 | F | 19.5 | 2 | 0 |
| 40 | MCI | 103 | F | 4.1 | 4 | 2 |
| 41 | MCI | 95 | M | 14 | 2 | 0 |
| 42 | MCI | 84 | M | 2.5 | 4 | 2 |
| **ID** | **Diagnosis^a^** | **Age (yr)^b^** | **Sex** | **PMI (hr)^c^** | **BST^d^** | **CERAD^e^** |
| 43 | CN | 87 | F | 3.9 | 2 | 0 |
| 44 | MCI | 90 | M | 10.9 | 4 | 2 |
| 45 | AD dementia | 82 | M | 7 | 4 | 2 |
| 46 | AD dementia | 91 | F | 10.8 | 5 | 3 |
| 47 | AD dementia | 93 | M | 3 | 4 | 1 |
| 48 | MCI | 87 | M | 21.2 | 3 | 2 |
| 49 | MCI | 81 | M | 17.6 | 2 | 0 |
| 50 | MCI | 90 | F | 12 | 2 | 2 |
| 51 | MCI | 97 | F | 20.8 | 5 | 2 |
| 52 | MCI | 88 | F | 6.2 | 3 | 3 |
| 53 | AD dementia | 96 | F | 3.6 | 3 | 2 |
| 54 | CN | 90 | M | 5.8 | 3 | 2 |
| 55 | CN | 79 | F | 17.4 | 2 | 1 |
| 56 | CN | 91 | M | 3.8 | 3 | 1 |
| 57 | AD dementia | 89 | F | 20.5 | 4 | 2 |
| 58 | CN | 81 | F | 23 | 1 | 2 |
| 59 | CN | 85 | M | 4 | 2 | 0 |
| 60 | MCI | 85 | M | 23.1 | 2 | 0 |
| 61 | MCI | 89 | F | 18.6 | 2 | 0 |
| 62 | MCI | 88 | M | 14 | 3 | 2 |
| 63 | AD dementia | 98 | F | 6 | 5 | 2 |
| 64 | CN | 61 | F | 1.6 | 0 | 0 |
| 65 | CN | 94 | F | 26.4 | 3 | 1 |
| 66 | MCI | 86 | M | 11 | 5 | 3 |
| 67 | AD dementia | 90 | F | 7 | 3 | 1 |
| 68 | MCI | 89 | F | 18 | 4 | 2 |
| 69 | CN | 86 | F | 18 | 4 | 0 |
| 70 | MCI | 90 | M | 28 | 4 | 1 |
| 71 | AD dementia | 94 | F | 43 | 5 | 3 |
| 72 | AD dementia | 89 | M | 7 | 4 | 2 |
| 73 | CN | 80 | M | 6 | n.d. | n.d. |
| 74 | MCI | 87 | F | 12 | 1 | 0 |
| 75 | AD dementia | 91 | F | 24 | 6 | 3 |
| 76 | CN | 91 | F | 21 | 2 | 1 |
| 77 | MCI | 90 | M | 18 | 4 | 2 |
| 78 | AD dementia | 85 | F | 16 | 6 | 3 |
| 79 | AD dementia | 92 | M | 4 | 4 | 3 |
| 80 | AD dementia | 84 | F | 21 | 6 | 3 |
| 81 | MCI | 91 | M | 12 | 5 | 0 |
| 82 | CN | 88 | F | 3.2 | 1 | 2 |
| 83 | CN | 89 | M | 16 | 3 | 2 |
| 84 | AD dementia | 89 | F | 13 | 4 | 2 |
| 85 | AD dementia | 87 | F | 44 | 5 | 3 |
| 86 | CN | 80 | M | 23 | 2 | 1 |
| **ID** | **Diagnosis^a^** | **Age (yr)^b^** | **Sex** | **PMI (hr)^c^** | **BST^d^** | **CERAD^e^** |
| 87 | CN | 93 | M | 11 | 3 | 1 |
| 88 | MCI | 79 | M | 26 | 2 | 0 |
| 89 | MCI | 101 | F | 11 | 3 | 2 |
| 90 | MCI | 75 | M | 11 | 2 | 2 |

^a^AD dementia= Alzheimer’s disease dementia, MCI = mild cognitive impairment, CN = cognitively normal.

^b^Age = age at death, yr = year.

^c^PMI = post-mortem interval of brain harvest, hr = hour.

^d^BST = Braak stage for neurofibrillary degeneration^3^, which was assigned based on a procedure previously described^4^. Specifically, 0 = AD-type neurofibrillary degeneration not present, 1 = Braak stage I, 2 = Braak stage II, 3 = Braak stage III, 4 = Braak stage IV, 5 = Braak stage V, 6 = Braak stage VI.

^e^CERAD = The consortium to establish a registry for Alzheimer's disease score for density of neocortical neuritic plaque^5^, which was assigned based on a procedure previously described^4^. Specifically, 0 = no neuritic plaques, 1 = sparse neuritic plaques, 2 = moderate neuritic plaques, 3 = frequent neuritic plaques.

n.d. = not determined.

**Supplementary Table S2 A statistical comparison of protein levels of the AD dementia patients and the individuals with MCI**

|  | **Mann-Whitney** | **Two-tailed, unpaired *t*-test^a^** | **Multiple linear regression^a,b^** |
| --- | --- | --- | --- |
| **Δtau314 (4F3)** | *p* = 0.45 | *p* = 0.32 | *p* = 0.29 |
| **Δtau314 (H1485)** | *p* = 0.29 | *p* = 0.28 | *p* = 0.24 |
| **T-tau** | *p* = 0.73 | *p* = 0.51 | *p* = 0.44 |
| **Δtau314 (4F3):T-tau** | *p* = 0.64 | *p* = 0.58 | *p* = 0.54 |
| **Δtau314 (H1485):T-tau** | *p* = 0.49 | *p* = 0.43 | *p* = 0.39 |
| **βIII-tubulin** | *p* = 0.17 | *p* = 0.83 | *p* = 0.99 |
| **Δtau314 (ELISA)** | *p* = 0.46 | *p* = 0.30 | *p* = 0.41 |
| **T-tau (ELISA)** | *p* = 0.74 | *p* = 0.98 | *p* = 0.74 |
| **Δtau314 (ELISA):T-tau (ELISA)** | *p* = 0.53 | *p* = 0.53 | *p* = 0.79 |
| **Casp2:total proteins** | *p* = 0.73 | *p* = 0.94 | *p* = 0.96 |

^a^The two-tailed, unpaired *t*-tests and multiple linear regressions were performed on the log-transformed outcomes.

^b^Multiple linear regression was used to analyze protein levels that were adjusted for age at death, sex, and post-mortem interval of brain tissue harvest of subjects.

**Supplementary references**

1 Ma, B. *et al.* PEAKS: powerful software for peptide de novo sequencing by tandem mass spectrometry. *Rapid Commun Mass Spectrom* **17**, 2337-2342, doi:10.1002/rcm.1196 (2003).

2 MacLean, B. *et al.* Skyline: an open source document editor for creating and analyzing targeted proteomics experiments. *Bioinformatics* **26**, 966-968, doi:10.1093/bioinformatics/btq054 (2010).

3 Braak, H. & Braak, E. Neuropathological stageing of Alzheimer-related changes. *Acta Neuropathol* **82**, 239-259 (1991).

4 Montine, T. J. *et al.* National Institute on Aging-Alzheimer's Association guidelines for the neuropathologic assessment of Alzheimer's disease: a practical approach. *Acta Neuropathol* **123**, 1-11, doi:10.1007/s00401-011-0910-3 (2012).

5 Mirra, S. S. *et al.* The Consortium to Establish a Registry for Alzheimer's Disease (CERAD). Part II. Standardization of the neuropathologic assessment of Alzheimer's disease. *Neurology* **41**, 479-486 (1991).
